# MCOLN1 is a ROS sensor in lysosomes that regulates autophagy

**DOI:** 10.1038/ncomms12109

**Published:** 2016-06-30

**Authors:** Xiaoli Zhang, Xiping Cheng, Lu Yu, Junsheng Yang, Raul Calvo, Samarjit Patnaik, Xin Hu, Qiong Gao, Meimei Yang, Maria Lawas, Markus Delling, Juan Marugan, Marc Ferrer, Haoxing Xu

**Affiliations:** 1 Department of Molecular, Cellular, and Developmental Biology, University of Michigan, 3089 Natural Science Building (Kraus), 830 North University, Ann Arbor, Michigan 48109, USA; 2 Collaborative Innovation Center of Yangtze River Delta Region Green Pharmaceuticals, College of Pharmaceutical Sciences, Zhejiang University of Technology, Hangzhou 310014, China; 3 National Center for Advancing Translational Sciences, National Institute of Health, 9800 Medical Center Drive, Rockville, Maryland 20850, USA; 4 The Department of Cardiology, Children's Hospital Boston, Enders 1350, 320 Longwood Avenue, Boston, Massachusetts 02115, USA

## Abstract

Cellular stresses trigger autophagy to remove damaged macromolecules and organelles. Lysosomes ‘host' multiple stress-sensing mechanisms that trigger the coordinated biogenesis of autophagosomes and lysosomes. For example, transcription factor (TF)EB, which regulates autophagy and lysosome biogenesis, is activated following the inhibition of mTOR, a lysosome-localized nutrient sensor. Here we show that reactive oxygen species (ROS) activate TFEB via a lysosomal Ca^2+^-dependent mechanism independent of mTOR. Exogenous oxidants or increasing mitochondrial ROS levels directly and specifically activate lysosomal TRPML1 channels, inducing lysosomal Ca^2+^ release. This activation triggers calcineurin-dependent TFEB-nuclear translocation, autophagy induction and lysosome biogenesis. When TRPML1 is genetically inactivated or pharmacologically inhibited, clearance of damaged mitochondria and removal of excess ROS are blocked. Furthermore, TRPML1's ROS sensitivity is specifically required for lysosome adaptation to mitochondrial damage. Hence, TRPML1 is a ROS sensor localized on the lysosomal membrane that orchestrates an autophagy-dependent negative-feedback programme to mitigate oxidative stress in the cell.

## Abstract (web summary)

Reactive oxygen species (ROS) damage cell components, necessitating their clearance through autophagy. Here, the authors show that ROS can induce autophagy by triggering TRPML1 to release Ca^2+^ from the lysosomal lumen, in turn activating the autophagy and lysosomal biogenesis regulator TFEB.

## Graphical abstract

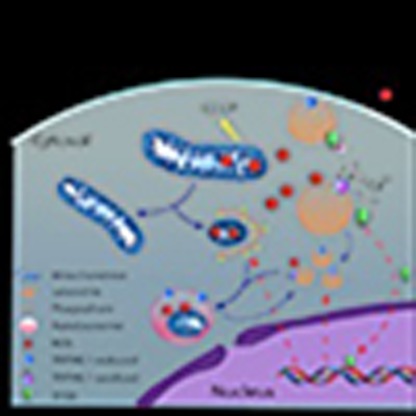

Reactive oxygen species (ROS) are generated mainly as byproducts of mitochondrial respiration, and their cytosolic levels are tightly controlled by multiple anti-oxidant mechanisms^1^. A regulatory imbalance can result in elevated ROS levels and oxidative stress, which are believed to underlie a variety of metabolic and neurodegenerative diseases, as well as aging^1^,^2^. Although high levels of ROS may additionally cause severe oxidative damage of proteins and lipids, a moderate ROS increase may serve as a sufficient ‘signal' to trigger autophagy and other cell-survival mechanisms^1^,^2^. Autophagy can target oxidized and damaged biomaterials selectively for lysosomal degradation^3^. Because unhealthy mitochondria may further augment ROS production, ROS-induced mitophagy is required for effective removal of excess ROS^1^,^4^,^5^. Hence, ROS and autophagy may constitute a negative feedback mechanism that mitigates oxidative stress and promotes cell survival.

Autophagy is a multi-step catabolic process that involves initiation (that is, phagophore formation), autophagosome biogenesis, lysosome biogenesis, autophagosome–lysosome fusion and lysosomal degradation^3^,^6^. ROS are known to induce autophagy, but the mechanisms underlying this induction are poorly understood^1^,^7^. ATG4, a cysteine protease and component of the cellular autophagy machinery, was recently identified as a direct target of ROS^8^. Oxidized ATG4 promotes lipidation of LC3 (a.k.a. ATG8), a process that is essential for autophagy initiation^8^. Because coping with prolonged oxidative stress may require sustained autophagy and sufficient lysosome supplies to achieve efficient autophagosome–lysosome fusion in an ongoing manner, it is hypothesized that the lysosome-participating steps of autophagy need to be upregulated in a coordinated manner by ROS signalling^1^,^6^,^7^,^9^,^10^. Indeed, given the essential role of lysosomes in autophagic clearance^6^, inadequate lysosomal function inevitably leads to metabolic and neurodegenerative diseases, even though autophagy induction is often elevated under these pathological conditions^3^,^6^.

Lysosomes are organelles that ‘host' important nutrient-sensitive molecules^11^. mTOR (the mechanistic target of rapamycin), described as the master regulator of growth, is a nutrient sensor that resides on lysosomal membranes^6^,^11^. Under starvation conditions, inhibition of mTOR results in a decrease in the phosphorylation of transcription factor (TF)EB, a master transcriptional regulator of both autophagy and lysosomal biogenesis^12^,^13^,^14^,^15^. Dephosphorylated TFEB proteins translocate rapidly to the nucleus from the cytosol and lysosomes, inducing or increasing the expression of a unique set of genes that are related specifically to autophagosome and lysosome biogenesis^13^,^15^. It is not yet known whether the mTOR–TFEB pathway regulates lysosome function in response to other cellular stresses.

Very recently, it was reported that TFEB-nuclear translocation can also be stimulated by lysosomal Ca^2+^ release and the Ca^2+^-dependent phosphatase calcineurin^9^. Lysosomal Ca^2+^ release is believed to be mediated by mucolipin 1 (MCOLN1 (a member of the transient receptor potential channel family), a.k.a. TRPML1), a key Ca^2+^-conducting channel on the lysosomal membrane that releases Ca^2+^ from the lumen into the cytosol^16^. However, it is unclear whether and how TRPML1 is activated by specific autophagy-inducing conditions, for example, oxidative stress and nutrient starvation.

Lysosomes are required for quality-control regulation of mitochondria, and oxidative stress is a common feature of lysosome storage diseases^16^. Recent studies suggest that mitochondria, the major source of endogenous ROS, are localized in close physical proximity to lysosomes^7^,^17^. Hence the lysosomal membrane is potentially an accessible and direct target of ROS signalling. Given that ROS reportedly regulate ion channels^18^, we hypothesize that lysosomal conductances, particularly through lysosomal Ca^2+^ channels such as TRPML1, may mediate ROS regulation of lysosomal function.

In the present study, we demonstrate a direct and specific activation of lysosomal TRPML1 channels by both exogenous oxidants and mitochondria-derived ROS in the endolysosomal patch-clamp recordings. This ROS-induced TRPML1 activation leads to lysosomal Ca^2+^ release, calcineurin-dependent TFEB-nuclear translocation and increases of LC3-II expression and autophagy. Genetic inactivation or pharmacological inhibition of TRPML1 impairs ROS-induced autophagy and blocks the clearance of damaged mitochondria and removal of excess ROS.

## Results

### Oxidants specifically activate lysosomal TRPML1 channels

**Figure 1:**
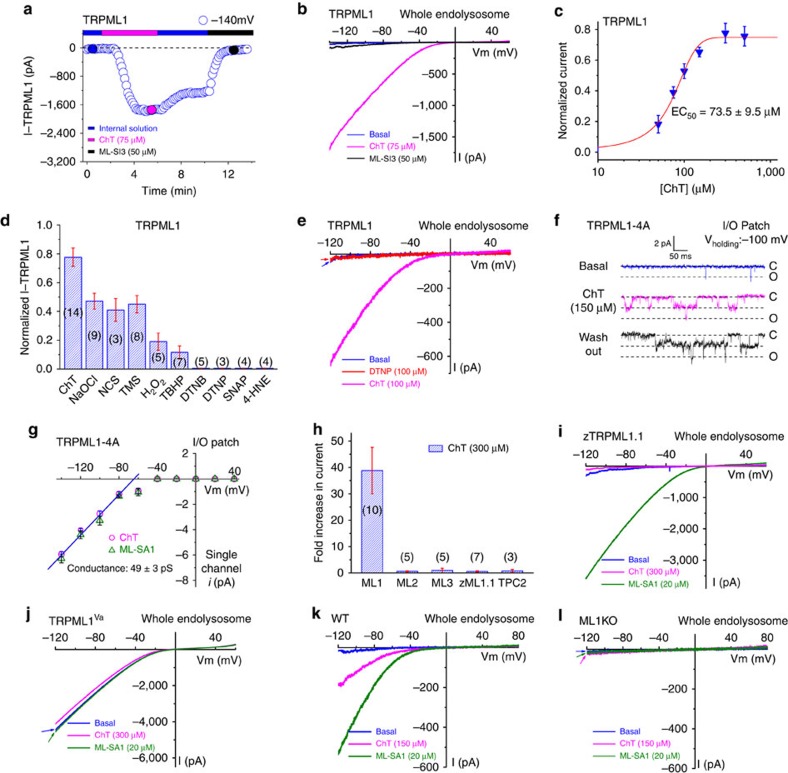
Direct and specific activation of lysosomal TRPML1 channels by ROS. (**a**) Representative time course of whole-endolysosome TRPML1-mediated currents (*I*_TRPML1_, open circles, measured at −140 mV) activated by bath application of ChT (75 μM), followed by a 4-min washout and then ML-SI3 (50 μM) application. *I*_TRPML1_ was recorded from an enlarged vacuole isolated from COS-1 cells overexpressing EGFP–TRPML1. The currents were elicited by repeated voltage ramps (−140 to +140 mV; 200 ms) with a 4-s inter-step interval. (**b**) Representative traces of basal (blue), ChT-activated (magenta) and ML-SI3-inhibited (black) *I*_TRPML1_ at the three time points indicated in **a**. Only a portion of the voltage protocol is shown; holding potential was 0 mV. (**c**) Dose-dependence of ChT activation (*n*=4–8 patches for each data point). (**d**) Oxidant-specific activation of *I*_TRPML1_. Active oxidants included ChT (150 μM), NaOCl (3 mM), *N*-chlorosuccinimide (NCS, 500 μM), thimerosal (TMS, 50 μM), H_2_O_2_ (10 mM) and *t*-butyl hydroperoxide (TBHP, 1 mM). Representative traces are in Supplementary Fig. 2. Other tested oxidants included cysteine-specific oxidants (DTNB and DTNP, both at 100 μM), an NO-donor (SNAP, 100 μM) and a reactive lipid (4-HNE, 300 μM). The effects of oxidants were normalized to that of ML-SA1 (20 μM). Numbers of patches tested for each oxidant are shown in brackets. (**e**) Representative traces of DTNP-insensitive (red), ChT-activated (magenta) *I*_TRPML1_. (**f**) ChT induced single-channel openings in an inside-out patch isolated from TRPML1-4A-expressing cells. (**g**) The single-channel conductance of ChT- and ML-SA1-activated *I*_TRPML1_. (**h**) ChT activated mTRPML1 specifically, but not mTRPML2, mTRPML3, zTRPML1.1 or mTPC2. Numbers of patches tested for each constructs are shown in brackets. (**i**) Insensitivity of zTRPML1.1 to ChT. (**j**) Insensitivity of whole-endolysosome TRPML1^Va^ currents to ChT. (**k**,**l**) ChT activated endogenous whole-endolysosome *I*_TRPML1_ in WT but not TRPML1 KO mouse macrophages. Data are presented as mean±s.e.m. in **c**,**d** and **h**.

We investigated the effects of oxidants on whole-endolysosome conductances in vacuolin-1-enlarged endolysosomes^19^,^20^ under a variety of experimental conditions in various cell types. In COS-1 cells overexpressing enhanced green fluorescent protein (EGFP)-tagged TRPML1 (Fig. 1a,b), whole-endolysosome TRPML1-mediated currents (*I*_TRPML1_) were activated strongly by bath application of chloramine T (ChT), a non-selective strong oxidant^21^,^22^. Unlike the responses to known TRPML1 agonists (that is, phosphatidylinositol 3,5-bisphosphate (PI(3,5)P_2_) and mucolipin-specific synthetic agonists (ML-SAs)^20^,^23^), activation by ChT persisted several minutes after washout (Supplementary Fig. 1). Sustained ChT-activated *I*_TRPML1_ was inhibited by the mucolipin-specific synthetic inhibitors (ML-SIs), but at concentrations higher than that are typically used to block TRPML1 (ref. 10). ChT activated *I*_TRPML1_ with an EC_50_ of 73.5±9.5 μM (mean±s.e.m., Fig. 1c), and with a potency comparable to those of PI(3,5)P_2_ and ML-SA1 (ref. 23; see Fig. 1c,d). ChT-, but not ML-SA1-, activation was abolished selectively by pretreatment with dithiothreitol (Supplementary Fig. 2), a reducing agent that is presumed to affect the overall redox status of the cell. Several other commonly used oxidants, including NaOCl, *N*-chlorosuccinimide, thimerosal, H_2_O_2_ and *t*-butyl hydroperoxide (TBHP) also readily, albeit less potently, activated *I*_TRPML1_ (Fig. 1d and Supplementary Fig. 3). Although relatively high concentrations (for example, 1–10 mM; also see Supplementary Fig. 9) of H_2_O_2_ were required for noticeable *I*_TRPML1_ activation *in vitro*, TBHP, a stable form of H_2_O_2_ (refs 18, 24), elicited *I*_TRPML1_ at much lower concentrations (<1 mM; see Supplementary Fig. 4). On the other hand, cysteine-modifying oxidants that are known to modulate several TRP channels, such as DTNP and DTNB^24^ (Supplementary Fig. 5), failed to activate *I*_TRPML1_ (Fig. 1d,e). Likewise, the NO-donor SNAP and the reactive lipid peroxidation intermediate 4-HNE^25^ did not affect *I*_TRPML1_ (Fig. 1d and Supplementary Fig. 6). Taken together, these results suggest that oxidants activate lysosomal TRPML1 channels via a distinct and novel mechanism.

We next investigated the mechanisms of oxidant-induced TRPML1 activation. Specifically, we tested whether the effect is direct in HEK293 cells expressing TRPML1-4A, a surface-expressing mutant TRPML1 channel^26^,^27^. In the inside-out, but not whole-cell, configuration, both ChT and NaOCl activated *I*_TRPML1-4A_ (Fig. 1f and Supplementary Figs 7 and 8), suggesting that the action is from the cytoplasmic side. ChT induced a dramatic and sustained increase in the channel open probability (NPopen) of single TRPML1 currents, which had a single-channel conductance comparable to the ML-SA1-activated currents (Fig. 1f,g). In contrast, TRPML1^Va^, a constitutively active mutant of TRPML1 (ref. 20), was insensitive to oxidants (Fig. 1j).

Unlike mouse and human TRPML1, oxidants failed to activate several other closely-related lysosome-localized channels, which include mouse TRPML2, mouse TRPML3, mouse two-pore channel-2 (TPC2; Fig. 1h and Supplementary Figs 7–9), and a zebrafish homologue of mammalian TRPML1 (zTRPML1.1; Fig. 1h,i and Supplementary Fig. 4). Consistently, oxidants activated endogenous TRPML1-like whole-endolysosomal currents in all mammalian cell types examined, including cultured primary wild-type (WT) macrophages (Fig. 1k), HeLa cells, HGT-1 parietal cell lines and HAP1 cells (Supplementary Fig. 10). In contrast, no measureable ChT-activated inward currents were detected in TRPML1 knockout (KO) mouse macrophages (Fig. 1l), suggesting that TRPML1 is the primary ROS-regulated conductance in the lysosome.

### Endogenous ROS trigger lysosomal Ca^2+^ release via TRPML1

**Figure 2:**
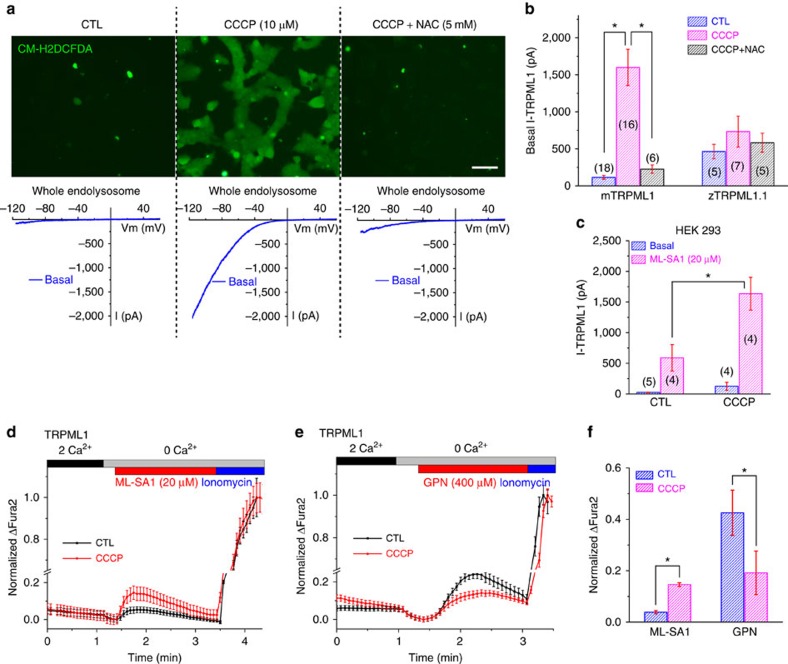
Endogenous mitochondrion-generated ROS activate lysosomal TRPML1 channels and Ca^2+^ release. (**a**) Upper panels: application of CCCP increased the fluorescence intensity of CM-H2DCFDA (green) versus the DMSO-treated control (CTL) group. The increase was inhibited by co-application of NAC (5 mM). Lower panels: representative traces of basal whole-endolysosomal currents under each condition (CTL, CCCP, CCCP+NAC) in TRPML1-expressing COS-1 cells. Scale bar, 50 μm. (**b**) Summary of CCCP pretreatment effects on basal *I*_TRPML1_ and *I*_zTRPML1.1_ from at least five patches for each experimental condition. Data are presented as mean±s.e.m. *P<0.05, ANOVA. Numbers of patches for each experimental condition are shown in brackets. (**c**) Effects of CCCP pretreatment on endogenous *I*_TRPML1_ in HEK293 cells (mean±s.e.m., *n*=4–5 patches for each treatment).*P<0.05, ANOVA. (**d**) Pretreatment of CCCP (10 μM) for 1 h increased ML-SA1-induced Ca^2+^ release measured by Fura-2 imaging in TRPML1-expressing HEK293 cells. (**e**) CCCP pretreatment reduced GPN-induced lysosomal Ca^2+^ release, which is presumed to reflect the lysosomal Ca^2+^ store. Mean values (±s.e.m.) are shown for >30 cells per coverslip. (**f**) Quantification of results shown in **d**,**e** from at least three independent experiments (mean±s.e.m.). *P<0.05, paired *t-*tests.

Because mitochondria are the primary source of endogenous ROS^1^,^4^,^5^, to evaluate the effect of endogenous ROS on TRPML1, we exposed cells to mitochondrial respiration inhibitors, for example, carbonyl cyanide m-chlorophenylhydrazone (CCCP) and rotenone, which are commonly used to induce ROS production, mitochondrial damage and subsequent mitophagy^28^,^29^. Following 1 h exposure to CCCP (5–10 μM) or rotenone (10 μM), intracellular ROS levels increased significantly (Fig. 2a and Supplementary Fig. 11), as reflected by visualization of CM-H2DCFDA, a ROS-sensitive fluorescent dye^29^. *N*-acetyl-cysteine (NAC), a commonly used membrane-permeable antioxidant^30^, abolished CCCP- or rotenone-induced increases in ROS levels (Fig. 2a,b and Supplementary Fig. 11). Because drugs like CCCP might produce ROS-independent effects due to mitochondrial depolarization/damage in addition to ROS production^28^,^29^, NAC sensitivity test was routinely performed in various cellular assays in the current study.

Remarkably, after CCCP pretreatment in TRPML1-expressing COS-1 cells, basal whole-endolysosome *I*_TRPML1_ increased significantly (Fig. 2a,b). Likewise, in non-transfected cells, ML-SA1-evoked endogenous *I*_TRPML1_ also increased (Fig. 2c and Supplementary Fig. 12). The CCCP-induced increase in basal *I*_TRPML1_, however, was largely abolished by NAC (Fig. 2a,b). In contrast, oxidant-insensitive *I*_zTRPML1.1_ was not affected by CCCP nor NAC pretreatment (Fig. 2b). Taken together, these results suggest that lysosomal TRPML1 is activated or sensitized by mitochondria-generated ROS in the cell.

Next, we investigated the effect of CCCP-generated mitochondrial ROS on lysosomal Ca^2+^ release. In COS-1 cells transfected with EGFP–TRPML1, ML-SA1-evoked lysosomal Ca^2+^ release^10^ was increased on CCCP (10 μM) pretreatment for 1 h (Fig. 2d–f). Under our experimental settings, CCCP, although known as a proton gradient de-coupler^28^,^29^, did not have a direct effect on *I*_TRPML1_ or lysosomal pH (Supplementary Figs 13 and 14). Consistently, acute treatment of CCCP did not directly evoke lysosomal Ca^2+^ release in GCaMP7–TRPML1-overexpressing cells (Supplementary Fig. 13). The constant Ca^2+^ release in CCCP-treated cells, presumably mediated by constitutive activity of TRPML1, is expected to continuously decrease Ca^2+^ stores in lysosomes. To test this possibility, glycyl-L-phenylalanine-beta-naphthylamide (GPN), a lysosome-specific substrate that causes channel-independent ‘leakage' of Ca^2+^, was employed to probe lysosome Ca^2+^ stores^31^. In contrast to ML-SA1-induced release, GPN-induced Ca^2+^ release was reduced in CCCP-treated cells (Fig. 2e,f). Note that in most other lysosomal Ca^2+^ studies, the ML-SA1 and GPN responses are positively correlated^10^,^23^. Together, these results are mostly consistent with an interpretation that in CCCP-treated cells, there is increased TRPML1-mediated Ca^2+^ release from lysosomes caused by high basal TRPML1 activity resulting from ROS elevation.

### TRPML1 is specifically required for ROS-induced autophagy

**Figure 3:**
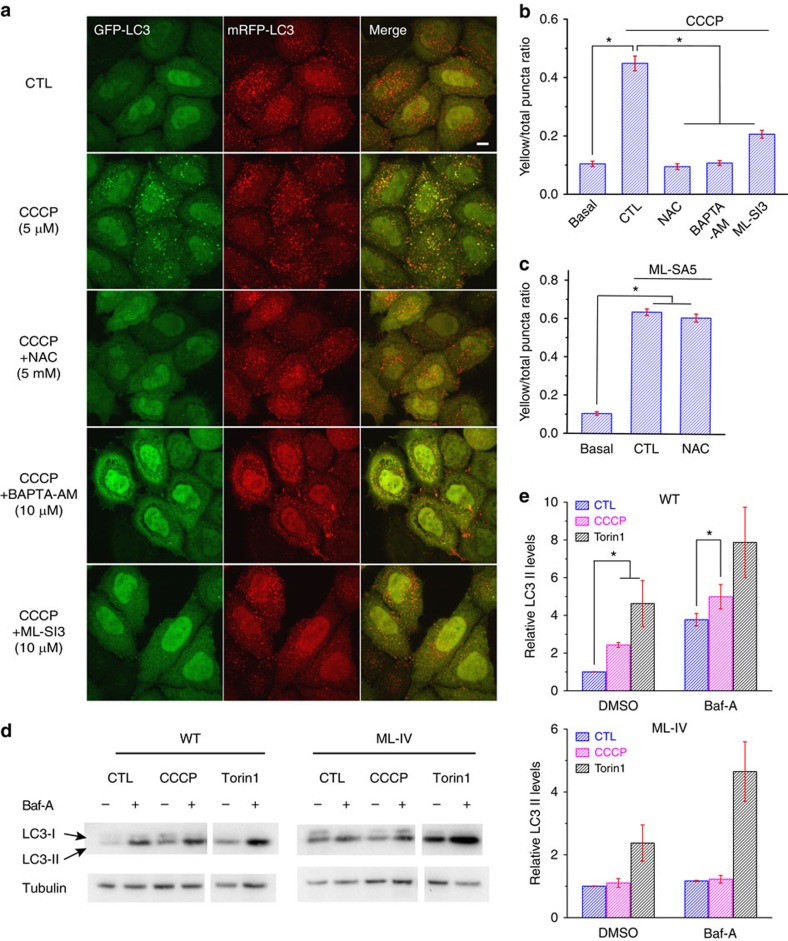
ROS-dependent autophagy induction requires Ca^2+^ and TRPML1. (**a**) In HeLa cells stably expressing mRFP–GFP–LC3, CCCP treatment (5 μM for 3 h) increased the formation of autophagosomes, ‘visualized' as mRFP^+^ GFP^+^ puncta. Co-treatment with NAC, BAPTA-AM, or ML-SI3 abolished the increases. Scale bar, 10 μm. (**b**) Quantification of various treatment conditions on CCCP-induced autophagosome formation (mean±s.e.m., *n*≥30 randomly-selected cells for each treatment). *P<0.05, ANOVA. (**c**) NAC did not affect ML-SA5-induced autophagosome formation. Means are shown with s.e.m. (*n*≥40 randomly-selected cells for each treatment). *P<0.05, ANOVA. (**d**) Western blot analysis of LC3-I and -II (arrows) protein expression in CCCP (10 μM, 3 h) -treated WT and ML-IV human fibroblasts. Torin 1 (1 μM) was used as a positive control to induce autophagy, and bafilomycin A1 (Baf, 0.5 μM) was used to inhibit lysosomal degradation. (**e**) Quantitative analysis of LC3-II levels under various experimental conditions shown in **d**. Data are presented as mean±s.e.m. (from at least three independent experiments); *P<0.05, paired *t-*tests.

CCCP-induced mitochondrial depolarization and/or ROS production is known to induce general autophagy, and mitophagy if there is extensive mitochondrial damage^5^. To investigate the role of TRPML1 in this process, we measured autophagic induction in HeLa cells stably expressing mRFP–GFP–LC3 (ref. 32). Because LC3-II is recruited specifically to phagophores and autophagosomes, and because of the pH sensitivity of the GFP signal, mRFP^+^ GFP^+^ and mRFP^+^ GFP^−^ puncta indicate non-acidified autophagosomes and acidified autolysosomes, respectively^3^. Three-hour exposure to CCCP (5 μM) led to a dramatic increase in autophagosomes, and this increase could be prevented by 3-methyladenine, an inhibitor of autophagy induction^3^ (Fig. 3a,b and Supplementary Fig. 15). Notably, under this experimental setting (that is, low dose of CCCP), there was only minimal initiation of mitophagy, assessed indirectly via the recruitment of PARKIN^4^ (Supplementary Fig. 16). Consistently, CCCP treatment also dramatically increased endogenous LC3-II levels in human fibroblasts (Fig. 3d,e). All these effects of CCCP were abolished by NAC (Fig. 3a,b and Supplementary Fig. 17), suggesting that CCCP-induced autophagy is mediated exclusively by ROS. Consistent with the hypothesis that ROS mediated the CCCP effect, H_2_O_2_ (100 μM, 3 h) treatment was sufficient to enhance autophagosome formation (Supplementary Fig. 18). Collectively, these results demonstrate that, in agreement with previous studies^1^,^7^, CCCP-mediated ROS generation induces autophagy.

Remarkably, CCCP- and H_2_O_2_-induced autophagosome formation was blocked by BAPTA-AM (membrane-permeable Ca^2+^ chelator^31^) or ML-SI3 (Fig. 3a,b and Supplementary Figs 17 and 18), suggesting that ROS induce autophagy via a Ca^2+^- and TRPML1-dependent mechanism. Furthermore, CCCP and H_2_O_2_ treatment failed to increase LC3-II levels in mucolipidosis IV patient-derived TRPML1 KO (ML-IV) fibroblasts (Fig. 3d,e and Supplementary Fig. 18), which exhibited high basal levels of LC3-II compared with WT cells (Supplementary Fig. 19). In both WT and ML-IV cells, LC3-II levels were readily increased by Torin 1, a potent inhibitor of mTOR that is commonly used to induce autophagy (Fig. 3d,e), suggesting that TRPML1 has a role specifically for ROS-mediated, but not for general autophagy induction. On the other hand, artificial activation of TRPML1 by the synthetic agonist ML-SA1, or the more potent agonists ML-SA3 and ML-SA5 (ref. 10), was sufficient to induce mRFP^+^ GFP^+^ LC3 puncta formation (Fig. 3c and Supplementary Fig. 18), but these effects were insensitive to NAC treatment (Fig. 3c and Supplementary Fig. 20). Furthermore, the induction effect of ML-SA5 on LC3-II levels was observed in WT but not ML-IV fibroblasts (Supplementary Fig. 20). Taken together, these results suggest that CCCP treatment may increase mitochondrial ROS, thereby activating TRPML1 to induce autophagy.

### TRPML1 is required for the clearance of damaged mitochondria

**Figure 4:**
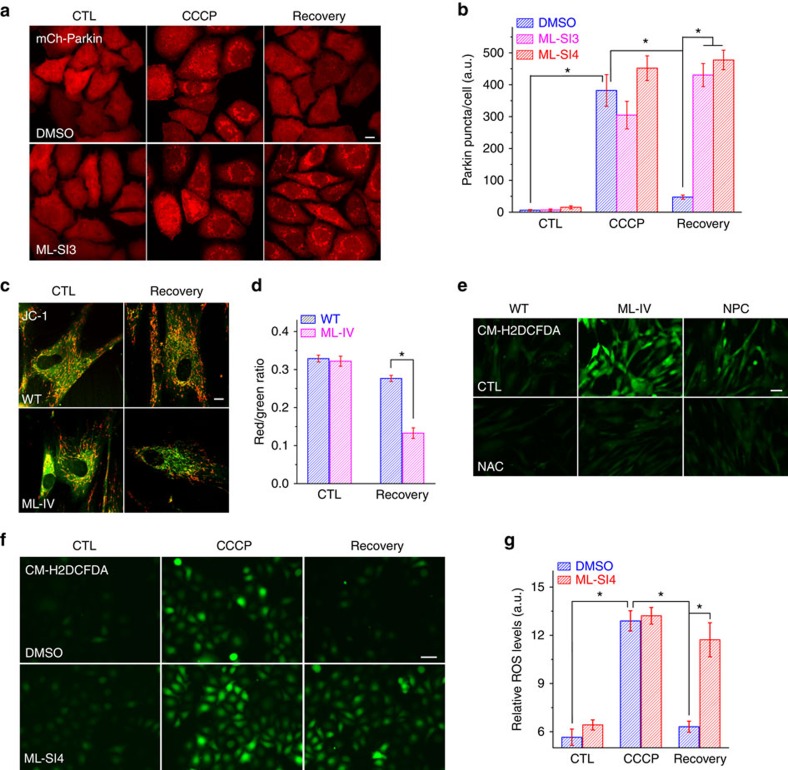
TRPML1 is required for autophagic clearance of damaged mitochondria and removal of excessive ROS. (**a**) Effects of ML-SI3 (10 μM) co-administration on the accumulation of PARKIN-positive puncta (red) induced by CCCP treatment (10 μM for 3 h) followed by 1 h recovery (without CCCP) in PARKIN stable cells. Scale bar, 10 μm. (**b**) Quantitative analysis of ML-SI3 and ML-SI4 effects on the clearance of PARKIN puncta. (**c**) Effects of CCCP treatment on mitochondrial membrane potential monitored by JC-1 fluorescent dyes in WT and ML-IV fibroblasts. After CCCP (10 μM for 3 h) treatment, removal of CCCP for 1 h led to repolarization (re-energization) of mitochondrial membrane potential (green, J-monomer; de-energized; red, J-aggregates; energized) in WT but not ML-IV cells. Scale bar, 10 μm. (**d**) The ratio of red to green fluorescence of JC-1 was quantified for >30 randomly-selected cells. Data are presented as mean±s.e.m. *P<0.05, ANOVA. (**e**) Basal ROS levels in WT, ML-IV and NPC fibroblasts. (**f**) Effect of ML-SI4 (10 μM) on ROS levels measured by CM-H_2_DCFDA (green) imaging in HeLa cells. Scale bar, 50 μm. (**g**) Quantification of results shown in **f**. Means are shown with s.e.m.; *P<0.05, ANOVA.

Sustained elevation of ROS levels can cause severe oxidative damage to mitochondria, triggering mitophagy^1^. ROS-induced mitophagy may facilitate the removal of damaged mitochondria and excess ROS products in the cell^1^. To investigate the role of TRPML1 in ROS-induced autophagic clearance of damaged mitochondria, we induced severe mitochondrial damage and fragmentation by exposing cells to high concentrations of CCCP for extended periods of time (for example, 10–20 μM for 3 h) (Supplementary Fig. 16). Using JC-1 fluorescence dye^33^ to monitor mitochondrial membrane potential, we found that such CCCP treatment resulted in rapid depolarization of mitochondria (data not shown). PARKIN proteins are known to be recruited specifically to the damaged mitochondria, which are then autophagocytosed and delivered to lysosomes for degradation^4^. In HeLa cells stably expressing mCherry–Parkin (PARKIN stable cells), PARKIN-positive puncta were increased significantly following CCCP treatment (Fig. 4a,b and Supplementary Fig. 21). Meanwhile, the increased LC3 puncta were often co-localized with PARKIN aggregates in GFP–LC3-overexpressing PARKIN stable cells, suggestive of increases in mitophagy (Supplementary Fig. 21). After CCCP washout, the majority of the PARKIN-positive puncta disappeared and most mitochondria returned to the repolarized state (Fig. 4a–d). The LC3-PARKIN co-localization, however, persisted during the recovery phase (Supplementary Fig. 21), suggesting a continuous removal of damaged mitochondria through active mitophagy. In contrast, acute inhibition of TRPML1 with ML-SI3 or ML-SI4 during the CCCP treatment phase was sufficient to block the disappearance of PARKIN, even though ML-SI3 or ML-SI4 alone did not induce the prolonged accumulation of PARKIN (Fig. 4a,b and Supplementary Fig. 21). Accordingly, CCCP-induced LC3-PARKIN co-localization (mitophagy) was significantly suppressed by ML-SI3 (Supplementary Fig. 21). Furthermore, JC-1 recovery was also blocked in ML-IV fibroblasts, compared with WT fibroblasts (Fig. 4c,d). Taken together, these results suggest that TRPML1 is required for ROS- and mitophagy-dependent clearance of damaged mitochondria.

Ongoing basal level of mitophagy may protect mitochondria from damage^7^. Although low-dose (for example, 5 μM) CCCP did enhance LC3 accumulation, no significant PARKIN aggregation could be observed (Supplementary Figs 16 and 23). Likewise, H_2_O_2_ treatment (100 μM for 3 h) did not produce significant PARKIN aggregation (Supplementary Fig. 22), although it did increase LC3 puncta formation and expression (Supplementary Fig. 18). However, in the presence of ML-SI3, low-dose CCCP treatment was sufficient to increase the number of PARKIN-positive puncta, although neither treatment alone had obvious effects (Fig. 4a and Supplementary Fig. 23). Hence, low levels of ROS may be sufficient to induce mitophagy, even though not detected experimentally by PARKIN recruitment. Taken together, these results suggest that TRPML1 may have a role in preventing the accumulation of damaged mitochondria.

### TRPML1 is required for efficient removal of excess ROS

ROS-induced mitophagy is hypothesized to remove excess ROS, preventing further oxidative damage to mitochondria^1^,^4^,^7^. As shown in Figs 2a and 4f, CCCP treatment increased ROS levels, which declined gradually on withdrawal of CCCP. However, in the presence of ML-SI3 or ML-SI4, the recovery of ROS levels was impeded (Fig. 4f,g). Furthermore, ROS levels were constitutively elevated in ML-IV cells, as well as in NPC (Niemann–Pick Type C) cells wherein TRPML1 is chronically inhibited^23^ (Fig. 4e). Collectively, these results suggest that TRPML1 activation is required for ROS-induced ROS removal, a negative feedback mechanism that is used by cells to circumvent oxidative stress.

### TRPML1 mediates ROS-induced TFEB activation

**Figure 5:**
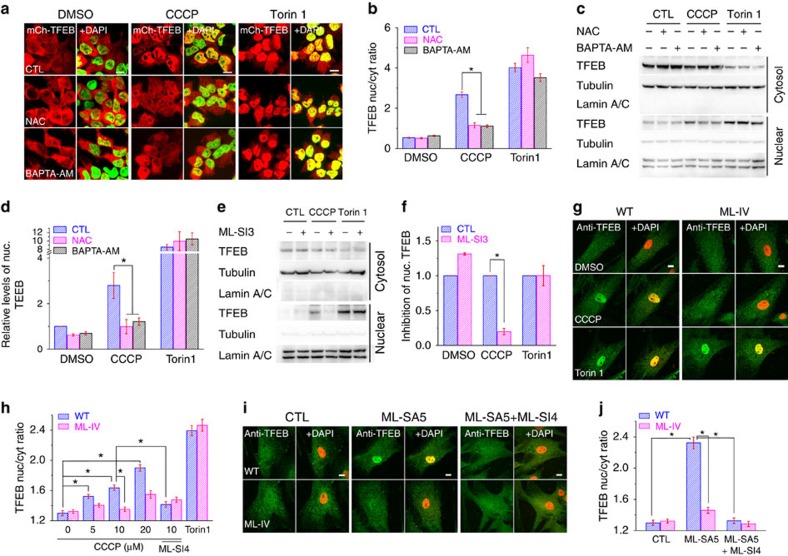
TRPML1 channel activity is required for ROS-induced TFEB-nuclear translocation. (**a**) Differential effects of BAPTA-AM and NAC on CCCP- and Torin-1-induced TFEB-nuclear translocation in HEK293 cells stably expressing mCherry–TFEB. Cells were treated with CCCP (5 μM) and Torin 1 (1 μM) for 1 h to induce TFEB-nuclear translocation. Nuclei were counterstained with DAPI (pseudo-colored in green). Scale bar, 10 μm. (**b**) Ratio of nuclear versus cytosolic TFEB (>100 cells per experimental condition). (**c**) Western blot analysis of cytosolic versus nuclear pools of TFEB proteins with and without CCCP treatment, and in the presence and absence of NAC and BAPTA-AM. Tubulin and Lamin are proteins abundant in the cytosolic and nuclear fractions, respectively. (**d**) Averaged effects of NAC or BAPTA-AM on CCCP-induced TFEB-nuclear translocation, based on multiple repeated experiments as shown in **c**. (**e**) Differential effects of ML-SI3 on CCCP- and Torin-1-induced TFEB-nuclear translocation, shown with western blot analyses of TFEB. (**f**) The quantitative effects of ML-SI3 on CCCP-induced TFEB-nuclear translocation based on the multiple repeated experiments as shown in **e**. (**g**) CCCP (10 μM for 1 h) induced accumulation of TFEB, detected by anti-human TFEB antibody, in the nuclei of WT, but not ML-IV cells. In contrast, Torin-1 induced TFEB-nuclear translocation in both WT and ML-IV cells. Nuclei were labelled with DAPI (pseudo-colored in red). Scale bar, 10 μm. (**h**) Average ratios of nuclear versus cytosolic TFEB immuoreactivity (>100 randomly-selected cells per experiment). ML-SI4 (10 μM) inhibited TFEB-nuclear translocation induced by CCCP (10 μM) treatment for 1 h. (**i**) The effects of ML-SA5 (1 μM for 1 h) on TFEB-nuclear translocation in the presence and absence of ML-SI4 (10 μM) in WT and ML-IV cells. Scale bar, 10 μm. (**j**) Average ratios of nuclear versus cytosolic TFEB immuoreactivity (>50 randomly-selected cells per experiment). All quantification data are presented as mean±s.e.m.; *P<0.05, paired *t-*test for western blots and ANOVA for all other comparisons.

We next investigated the mechanisms by which TRPML1 activation leads to enhanced autophagy induction and mitophagic clearance. TFEB regulates biogenesis of both autophagosomes and lysosomes^34^. Recent evidence suggests that TRPML1 and TFEB may form a positive-feedback loop in regulating autophagy^9^,^10^. In HEK293 cells stably expressing mCherry–TFEB (TFEB stable cells), we found that a mild increase in ROS levels due to CCCP treatment (5 μM for 1 h) was sufficient to induce TFEB-nuclear translocation (nuclear to cytosol ratio increased from 0.54±0.02 to 2.67±0.14 (mean±s.e.m., *n*=3 experiments for each treatment); see Fig. 5a,b). Likewise, endogenous TFEB in human fibroblasts was also activated (that is, underwent nuclear translocation) in response to CCCP administration in a dose-dependent manner (Fig. 5g,h and Supplementary Fig. 24). The specificity of TFEB immunoreactivity was confirmed in TFEB KO HeLa cells (Supplementary Fig. 25) generated by the CRISPR/Cas9 system^35^. In addition, nuclear isolation analyses also demonstrated that CCCP treatment induced nuclear translocation of TFEB proteins (Fig. 5c,d) in TFEB stable cells. In both WT and PARKIN stable cells, CCCP (5–20 μM) activated TFEB (Supplementary Fig. 26), even in the presence of NOX inhibitors, which block mitochondria-independent ROS-generating NADPH oxidases^36^ (Supplementary Fig. 27). Likewise, rotenone (10 μM for 2 h) and H_2_O_2_ treatment (50 μM for 1 h) were also sufficient to induce TFEB translocation in WT HeLa cells (Supplementary Figs 28 and 29). Remarkably, in all cases, TFEB-nuclear translocation, induced by CCCP, rotenone or H_2_O_2_, was largely blocked by NAC, BAPTA-AM or ML-SI3 (Fig. 5a–f and Supplementary Figs 23, 28 and 29).

ROS levels are reportedly elevated during starvation^8^, a condition that activates TFEB and promotes autophagy^9^. Indeed, short-term (4–6 h) starvation increased ROS levels and caused TFEB-nuclear translocation (Supplementary Figs 30 and 31). However, starvation-induced TFEB activation was not blocked by NAC or its more potent derivative NACA, nor by ML-SI3 (Supplementary Figs 30 and 31). These results suggest that starvation may induce TFEB activation via mechanisms that do not require TRPML1. Consistently, 4-h starvation induced TFEB-nuclear translocation in both WT and ML-IV human fibroblasts (Supplementary Fig. 31). Collectively, these results suggest that whereas ROS activation of TRPML1 is sufficient to activate TFEB, additional TRPML1-independent mechanisms may be responsible for starvation-induced TFEB activation.

CCCP-mediated TFEB translocation was largely abolished in ML-IV fibroblasts (Fig. 5g,h and Supplementary Fig. 24), as well as in WT cells treated with ML-SI3 or ML-SI4 (Fig. 5e–h). Furthermore, ML-SA5 alone was sufficient to cause TFEB-nuclear translocation in WT cells (Fig. 5i,j and Supplementary Fig. 32). This ML-SA5-induced TFEB translocation was sensitive to BAPTA-AM, but not NAC (Supplementary Fig. 32). Neither CCCP nor ML-SA5 affected the activity of mTOR, as reflected by levels of phosphorylated S6K, a primary mTOR substrate^11^ (Supplementary Fig. 33). On the other hand, Torin-1-induced TFEB-nuclear translocation, observed in both WT and ML-IV cells (Fig. 5g, h and Supplementary Fig. 24), was not sensitive to BAPTA-AM or ML-SI3 (Fig. 5a–f). Hence, TRPML1 and lysosomal Ca^2+^ appear to play a specific role in ROS-induced, but not the general mTOR-inhibition-mediated, autophagy. More strikingly, in PARKIN stable cells, when TFEB translocation was blocked by ML-SI3, obvious aggregation of PARKIN-positive puncta was observed even with a low-dose CCCP treatment (Supplementary Fig. 23). Consistent with the reported role of calcineurin in TFEB activation^9^, we found that TFEB translocation induced by CCCP was also largely blocked by calcineurin inhibitors, FK506 and Cyclosporin A (Supplementary Fig. 34). Taken together, these results suggest that TRPML1-dependent activation of TFEB plays a crucial role in ROS-induced autophagy and mitophagy.

### ROS-induced lysosome biogenesis requires TRPML1

**Figure 6:**
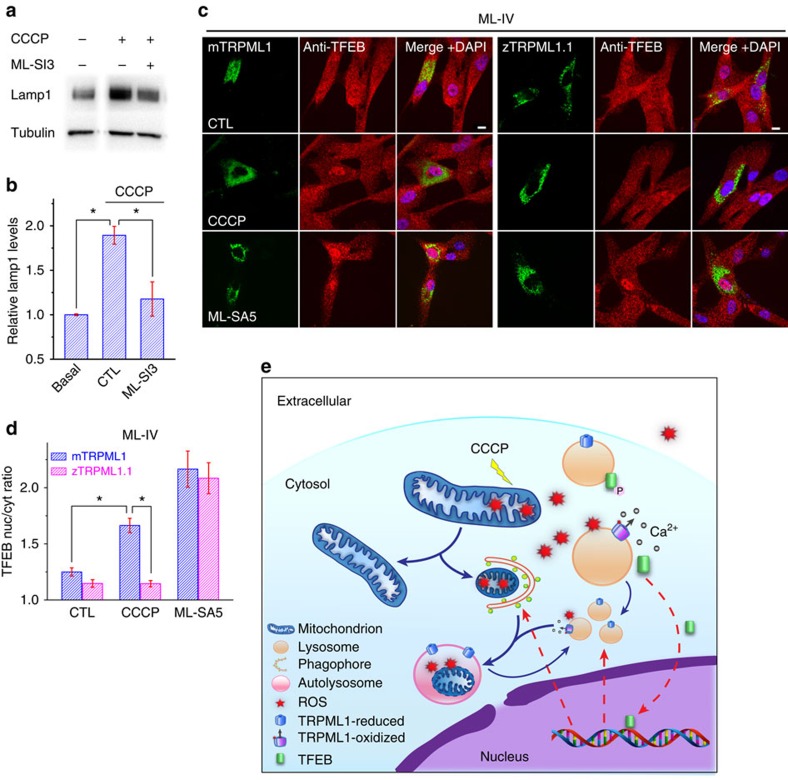
ROS sensitivity of TRPML1 is required for TFEB activation by mitochondrial ROS. (**a**) Effect of ML-SI3 on Lamp1 expression 7 h after CCCP treatment (5** **μM for a duration of 1 h). Note that there was a progressive increase in Lamp1 expression levels following CCCP withdrawal (see Supplementary Fig. 35). (**b**) Quantification of **a** from three independent experiments (mean±s.e.m.); *P<0.05, paired *t-*test. (**c**) Rescue of CCCP-induced TFEB-nuclear translocation in ML-IV fibroblasts by transfection of mTRPML1, but not zTRPML1.1 constructs. ML-SA5 induced TFEB-nuclear translocation in ML-IV cells transfected with either mTRPML1 or zTRPML1.1. Scale bar, 10 μm. (**d**) Quantification of experimental results as shown in **c**. Data are presented as mean±s.e.m.; *P<0.05, ANOVA. (**e**) A working model to illustrate the role of TRPML1 in ROS-induced TFEB activation and autophagy. An increase in mitochondrial ROS (for example, by CCCP-mediated mitochondrial depolarization) may activate TRPML1 channels on the perimeter membranes of lysosomes, inducing lysosomal Ca^2+^ release of that activates calcineurin. Subsequently, Ca^2+^-bound calcinurin dephosphorylates TFEB, which is otherwise kept in its phosphorylated form by the nutrient-sensitive lysosome-localized mTOR kinase^15^. Nucleus-localized TFEB then activates the transcription of a unique set of genes related to autophagy induction, autophagosome biogenesis and lysosome biogenesis. Lysosomal Ca^2+^ release may also directly promote lysosome reformation/biogenesis^9^. Subsequently, autophagy is promoted to facilitate clearance of damaged mitochondria and removal of excessive ROS.

Because TFEB is a transcriptional regulator of lysosome biogenesis^11^, we next investigated the roles of ROS and TRPML1 in lysosome biogenesis. Increased expression of lysosomal housekeeping gene Lamp1 was employed as a read-out of lysosome biogenesis^10^,^16^. Upon CCCP (5 μM) treatment for 1 h, which produced a modest increase in ROS levels, expression of Lamp1 increased gradually in HeLa cells (Supplementary Fig. 35). Therefore, a brief ROS burst is sufficient to trigger lysosome biogenesis. However, the increase was largely attenuated by ML-SI3 (Fig. 6a,b) and also diminished in TFEB KO cells (Supplementary Fig. 35), suggesting that TRPML1 is required for ROS-induced lysosome biogenesis through TFEB activation.

### ROS sensitivity of TRPML1 in CCCP-induced TFEB activation

To test the hypothesis that CCCP-induced TFEB activation requires redox regulation of TRPML1, we transfected ML-IV fibroblasts with ROS-sensitive mTRPML1 or ROS-insensitive zTRPML1.1 constructs, respectively. Unlike untransfected ML-IV cells, mTRPML1-expressing ML-IV cells displayed clear TFEB-nuclear translocation in response to CCCP (Fig. 6c,d). In contrast, in zTRPML1.1-expressing ML-IV cells, no significant CCCP-induced TFEB translocation was observed (Fig. 6c,d). On the other hand, ML-SA5 induced robust TFEB-nuclear translocation in both mTRPML1- and zTRPML1.1-expressing ML-IV cells. Therefore, redox regulation of TRPML1 is specifically required for ROS-induced TFEB activation and autophagy.

## Discussion

In the current study, we identified the missing links between ROS and autophagy: lysosomal Ca^2+^, TRPML1 and TFEB (see Fig. 6e). Our results suggest a model wherein an elevation of ROS levels (for example, due to mitochondrial damage) leads to TRPML1 activation and lysosomal Ca^2+^ release. Lysosomal Ca^2+^, acting via calcineurin-mediated dephosphorylation^9^, induces TFEB-nuclear translocation, promoting both autophagosome biogenesis and lysosome biogenesis. Lysosomal Ca^2+^ may also directly regulate lysosome biogenesis and autophagosome–lysosome fusion^16^. The subsequent increase in autophagic flux may facilitate removal of damaged mitochondria and restoration of redox homeostasis. Hence TRPML1 may serve as a ROS sensor in the lysosome that regulates an autophagy-dependent negative-feedback mechanism essential for cellular redox homeostasis.

Although the connection between ROS and autophagy has been well-documented^1^,^7^, the mechanisms by which ROS promote autophagy are largely unknown. Several TFs, including NRF2 and FOXO3, are known to become active under oxidative stress conditions^7^. Recently, ATG4, a cytosolic negative regulator of LC3/ATG8, was identified as a direct ROS target that provides a rapid switch mechanism for autophagy induction^8^. TRPML1 is activated rapidly by exogenous oxidants in isolated lysosomes, or in excised patches containing TRPML1 re-directed to the plasma membrane. Hence, it is very likely that direct oxidation of TRPML1 occurs, and such oxidation favours the channel adopting a ‘Va-like' constitutively-open state^20^. Because autophagy is a multi-step process^3^, there may be multiple ROS sensors involved in regulating autophagy. Notwithstanding, in our experimental paradigm, in which endogenous mitochondrial ROS are generated to induce autophagy, autophagy induction is almost completely blocked by Ca^2+^ chelators and TRPML1 inhibitors. The robustness of these results suggests that TRPML1 may play a pivotal role in autophagy regulation.

Our findings are consistent with the reported role of Ca^2+^ in autophagosome biogenesis^37^. However, in light of the observations that increasing autophagosome formation alone does not result in an increase in autophagic flux, ROS-dependent mechanisms that promote lysosome biogenesis and function must exist. Given the established roles of TRPML1 and TFEB in lysosomal trafficking and biogenesis^9^,^10^,^16^, our identification of TRPML1 as a lysosomal ROS sensor has revealed a unique ROS-regulated paradigm in which both initiation and maturation of autophagy are coordinated.

Lysosomal membranes are the focal sites of multiple nutrient-sensitive kinases that regulate autophagy, including mTOR and AMPK^11^. It may not be coincidental that TRPML1, acting as a major autophagy-regulating ROS sensor, is also localized on the lysosomal membrane. TRPML1, a protein implicated in lysosome reformation and autophagosome–lysosome fusion, is upregulated in response to cellular stress, such as nutrient starvation^10^,^16^. TRPML1 and TFEB/TFE3 may form a positive-interaction loop to regulate lysosome function and autophagy^9^,^10^,^16^. TRPML1's direct role in stress-sensing makes TRPML1 and TFEB well-situated to play important roles in lysosome adaptation to environmental cues^10^. However, TFEB can also be activated by TRPML1-independent pathways, such as by mTOR inhibition and PIKfyve inhibition^10^. Hence TRPML1 appears to be uniquely positioned to respond to certain stress pathways, including ROS signalling.

ROS regulation of TRPML1 may also affect other lysosome-related functions. ROS can be generated within lysosomes when heavy metal ions (for example, iron) catalyse the Fenton reaction, thereby generating free radicals^38^. Indeed, TRPML1's iron permeability^39^ and ROS sensitivity are well-suited to constitute a negative feedback mechanism to regulate redox homeostasis in the lysosome, preventing lysosomal membrane permeability and lysosomal cell death^16^. Likewise, ROS regulation of TRPML1 may also be involved in membrane repair and phagocytic killing of bacteria, both of which are known to involve oxidant signalling^40^.

Under physiological conditions, a mild increase in ROS levels may act as a ‘survival' signal triggering autophagy for the ‘quality control' purpose, whereas a large increase in ROS levels may produce severe oxidative damage and stress and serve as a ‘death' signal^7^. Hence before the ‘death threshold' is reached, ROS can signal both autophagy induction and lysosome biogenesis, promoting autophagic flux. Supra-threshold ROS, however, may cause lysosomal membrane permeability, lysosomal dysfunction and autophagic failure. For example, the excessive and sustained surge in ROS that occurs during cardiac ischemia–reperfusion may actually inhibit autophagy, contributing to cardiomyocyte death^41^. However, under pathological hyper-sensitive conditions, in which lysosome function and autophagy are not functioning appropriately, even a mild increase in ROS levels may cause oxidative stress. For example, when the TRPML1–TFEB pathway is inactive or compromised, as is seen in several lysosomal storage diseases, cells exhibit mitochondrial fragmentation, elevation of basal ROS levels and oxidative stress^16^. On the other hand, when TRPML1–TFEB activity is upregulated, oxidative stress is mitigated^9^,^10^,^16^,^42^. Hence, the TRPML1–TFEB pathway may represent a potential therapeutic target by which preemptive modulation of oxidative stress may alleviate symptoms in patients with lysosomal storage diseases and neurodegenerative diseases characterized by excess ROS.

## Methods

### Molecular biology

TRPML1 mutants were constructed with a site-directed mutagenesis kit (Qiagen) using mouse TRPML1 as the template. GCaMP7–TRPML1 was generated by inserting the full-length GCaMP7 between the HindIII and EcoRI sites at a pcDNA6–mTRPML1 construct^23^. The mCherry–PARKIN construct was provided by Dr Richard Youle through Addgene^4^. All constructs were confirmed by DNA sequencing.

### Mammalian cell culture

COS-1 and HEK-293T were cultured in a 1:1 mixture of DMEM and Ham's F12 (DF12) media with 10% fetal bovine serum (FBS). HeLa and HAP1 cells were maintained in DMEM and IMDM, respectively, both with 10% FBS. Lipofectamine 2000 (Invitrogen) was used for the transfection of above cells. Human skin fibroblast cell lines from a mucolipidosis IV (TRPML1 KO) patient (clone GM02048) and a healthy control (clone GM05659) were obtained from the Coriell Institute for Medical Research (NJ, USA). Fibroblasts were transfected with a Neon electroporation kit (Invitrogen). Culture media were refreshed 18–24 h post-transfection, and cells were imaged 48 h post-transfection to allow sufficient recovery time following transfection.

### Stable cell lines

The mCherry–PARKIN stable cell line was generated in HeLa cells under the selection of 500 mg l^−1^ Geneticin (G418, Invitrogen). The mCherry–TFEB stable cell line was generated using the Flip-In T-Rex 293 cell line (Invitrogen) under blasticidin selection. GFP–mRFP–LC3 and GFP–TFEB stable cell lines were kindly provided by Drs David Rubinsztein^32^ and Shawn M. Ferguson^13^, respectively. Unless otherwise indicated, all cell lines were maintained in DMEM medium supplemented with 10% Tet-free FBS at 37 °C in a humidified 5% CO_2_ incubator.

### CRISPR KO

TFEB CRISPR KO cells were generated in HeLa cells using the CRISPR/Cas9 system^43^. The TFEB sequence in the second exon 5′-GACGGGGGTATTGATGGCCG-3′ (TFEB–sgRNA) was targeted with pSpCas9 (BB)-2A-puro vector (Addgene). HeLa cells were then transfected with TFEB–sgRNA-expressing vector using Lipofectamine 2000 and selected in the presence of 5 μg ml^−1^ puromycin for 48 h. After single cell clones were established, their genomic DNAs were sequenced to confirm the intended genetic disruptions.

### Confocal imaging

For TFEB and TFE3 immunofluorescence detection, cells were grown on glass coverslips and then fixed with 4% paraformaldehyde and permeabilized with 0.3% Triton X-100 after treatments. The cells were then blocked with 1% bovine serum albumin in phosphate buffered saline (PBS). Endogenous TFEB and TFE3 were recognized by incubating cells with anti-TFEB (1:200; Cell Signaling Technology) or anti-TFE3 antibody (1:1,000 Sigma) at 4 °C overnight. Cells were then washed four to five times with PBS and incubated with anti-rabbit secondary antibodies conjugated to Alexa Fluor 568 or 488 (Invitrogen) for 1 h. After three washes with PBS, coverslips were mounted on the slides with Fluoromount-G (Southern Biotech). Images were acquired with an Olympus Spinning-Disk Confocal microscope.

### Western blotting

Cells were lysed with ice-cold RIPA buffer (Boston BioProducts) in the presence of 1 × protease inhibitor cocktail (Sigma), 1 mM NaF and 1 mM Na_3_VO_4_. Total cell lysates were mixed with 2 × SDS-loading buffer and were boiled at 95 °C for 10 min. Protein samples (10–100 μg) were then loaded and separated on 4–12% gradient SDS–PAGE gels (Invitrogen) and transferred to polyvinylidene difluoride membranes. The membranes were blocked for 1 h with 1% bovine serum albumin in PBS supplemented with 0.1% Tween 20 and were incubated with antibodies against GFP (1:10,000; Covance, MMS-118P), LC3 (1:2,000; Sigma, L8918), Lamin A/C (1:1,000; Cell Signaling Technology, 4777), Lamp1 (1:1,000; Developmental Studies Hybridoma Bank, H4A3), γ-tubulin (1:4,000; Sigma, T5326), TFEB (1:1,000; Cell Signaling Technology, 4240) and TFE3 (1:4,000; Sigma, HPA023881), respectively. Bound antibodies were detected using horseradish peroxidase-conjugated anti-rabbit (65-6120) or anti-mouse (62–6520) secondary antibodies (1:5,000, Invitrogen) and enhanced chemiluminescence reagent (Amersham Pharmacia Biotech). Band intensities were quantified in Image J software. Full versions of all blots are shown in Supplementary Fig. 36.

### Ca^2+^ imaging

Fura-2 Ca^2+^ imaging was carried out in cells loaded with 5 μM Fura-2 AM (Invitrogen) at 37 °C for 1 h, as described previously^23^. In brief, fluorescence, at two excitation wavelengths, F_340_ and F_380,_ was recorded with an EasyRatioPro system (PTI). Fura-2 ratios (F_340_/F_380_) were used to monitor changes in intracellular [Ca^2+^]. Lysosomal Ca^2+^ release was measured under a zero-Ca^2+^ external solution, which contained 145 mM NaCl, 5 mM KCl, 3 mM MgCl_2_, 10 mM glucose, 1 mM EGTA and 20 mM HEPES (pH 7.4); free [Ca^2+^]_o_<10 nM (estimated with Maxchelator software http://maxchelator.stanford.edu/).

GCaMP imaging was performed in HeLa cells transfected with GCaMP7–TRPML1, a lysosome-targeted genetically-encoded Ca^2+^ sensor^23^. The fluorescence intensity at 488 nm (F488) was recorded at 37 °C with the spinning-disk confocal live-imaging system, which included an Olympus IX81 inverted microscope, a × 60 or × 100 objective (Olympus), a CSU-X1 scanner (Yokogawa), an iXon EM-CCD camera (Andor) and MetaMorph Advanced Imaging acquisition software v.7.7.8.0 (Molecular Devices).

### ROS and lysosomal pH imaging

ROS levels were detected with a CM-H2DCFDA dye assay (Invitrogen). Briefly, cells were incubated with 2.5–5 μM CM-H2DCFDA in the culture media without FBS at 37 °C for 30 min, and then recovered in the complete media for 10 min before imaging. For non-quantitative estimation of lysosomal luminal pH, cells were incubated with 50 nM LysoTracker Red DND-99 (Invitrogen) in complete culture medium for 15 min before imaging. The fluorescence was visualized with a DP71 camera (Olympus) mounted on an Olympus IX-71 inverted microscope. Images were captured at 20 × magnification with DPController software. The fluorescence intensity was quantified with the ImageJ software (NIH).

### Mitochondrial membrane potential measurement

Human fibroblasts were incubated with 1 μM JC-1 (Invitrogen) in complete culture medium at 37 °C for 30 min before imaging. The fluorescence was detected at 520 nm for J-monomer and 600 nm for J-aggregates (excitation wavelength=488 nm) by a Leica confocal microscope.

### Whole-endolysosome electrophysiology

Isolated endolysosomes were subjected to whole-endolysosomal electrophysiology by a modified patch-clamp method^19^,^20^. Briefly, cells were treated with 1 μM vacuolin-1 overnight to selectively increase the size of late endosomes and lysosomes^44^. Enlarged vacuoles were released into the dish by mechanical disruption of the cell membrane with a fine-tip glass electrode. Unless otherwise indicated, vacuoles were bathed continuously in an internal (cytoplasmic) solution containing 140 mM K^+^-Gluconate, 4 mM NaCl, 1 mM EGTA, 2 mM Na_2_-ATP, 2 mM MgCl_2_, 0.39 mM CaCl_2_, 0.1 mM GTP and 10 mM HEPES (pH adjusted with KOH to 7.2; free [Ca^2+^]_i_≈100 nM). The pipette (luminal) solution contained 145 mM NaCl, 5 mM KCl, 2 mM CaCl_2_, 1 mM MgCl_2_, 10 mM HEPES, 10 mM MES and 10 mM glucose (pH adjusted to 4.6 with NaOH). The whole-endolysosome configuration was achieved as described previously^19^. In brief, after formation of a gigaseal between the patch pipette and an enlarged endolysosome, voltage steps of several hundred millivolts with a millisecond duration were applied to break into the vacuolar membrane^19^. All bath solutions were applied via a fast perfusion system that produced a complete solution exchange within a few seconds. Data were collected via an Axopatch 2A patch-clamp amplifier, Digidata 1440 and processed with pClamp 10.0 software (Axon Instruments). Whole-endolysosome currents were digitized at 10 kHz and filtered at 2 kHz. All experiments were conducted at room temperature (21–23 °C) and all recordings were analysed in pCLAMP10 (Axon Instruments) and Origin 8.0 (OriginLab).

### Whole-cell and inside-out patch-clamp electrophysiology

Whole-cell recordings were performed with pipette electrodes (resistance 3–5 MΩ) filled with (in mM): (1) 133 Cs methanesulfonate, 4 NaCl, 10 EGTA, 2 Na_2_-ATP, 2 MgCl_2_ and 20 HEPES (pH 7.2, adjusted with CsOH; free [Ca^2+^]_i_<10 nM); or (2) 140 K-gluconate, 4 NaCl, 1 EGTA, 2 MgCl_2_, 0.39 CaCl_2_ and 20 HEPES (pH 7.2; free [Ca^2+^]_i_∼100 nM). The standard extracellular bath solution (Tyrode's solution) contained (in mM): 153 NaCl, 5 KCl, 2 CaCl_2_, 1 MgCl_2_, 20 HEPES and 10 glucose (pH 7.4). For excised inside-out patch recordings, pipette electrodes with 1–2 MΩ resistance were used. The bath solution and pipette solution were the same as those used for whole-endolysosome recordings.

### Reagents

The following reagents were purchased: ML-SA1 (Princeton BioMolecular Research Inc), Torin 1 (Tocris), NAC (Sigma), CCCP (Sigma), H_2_O_2_ (Sigma), ChT (Sigma), NSC (Sigma), DTNP (Sigma), 4-HNE (Caymen), Ionomycin (Sigma), BAPTA-AM (Invitrogen) and vacuolin-1 (Calbiochem). ML-SA and ML-SI compounds were identified from a Ca^2+^-imaging-based high-throughput screening conducted at NIH/NCATS Chemical Genomics Center (NCGC; see https://pubchem.ncbi.nlm.nih.gov/bioassay/624414#section=Top). ML-SA and ML-SI compounds are available upon request.

### Data analysis

Data are presented as mean±s.e.m. Statistical comparisons of confocal images were performed with analyses of variance (ANOVA). Protein expression levels were compared using paired *t*-test. A *P-*value<0.05 was considered statistically significant.

### Data availability

The data that support the findings of this study are available from the corresponding author upon request.

## Additional information

**How to cite this article:** Zhang, X. *et al.* MCOLN1 is a ROS sensor in lysosomes that regulates autophagy. *Nat. Commun.* 7:12109 doi: 10.1038/ncomms12109 (2016).

## Supplementary Material

Supplementary Information Supplementary Figures 1-36
