# Supplementary material for: MCOLN1 is a ROS sensor in lysosomes that regulates autophagy
Source: Nat Commun. 2016 Jun 30;7:12109. doi: 10.1038/ncomms12109 (PMC4931332; doi:10.1038/ncomms12109)
Supplement: Supplementary Information — Supplementary Figures 1-36 [file ncomms12109-s1.pdf]

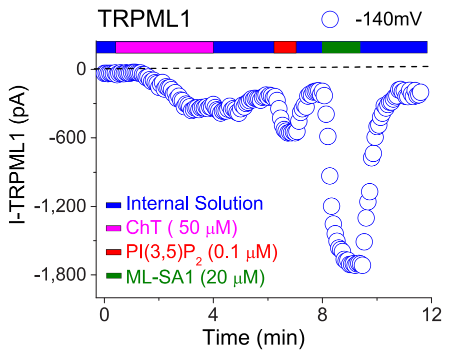

Supplementary Figure 1. Time courses of TRPML1 activation by ChT, PI(3,5)P2 and ML-SA1. In comparison with PI(3,5)P2 and ML-SA1, ChT application led to sustained activation of whole-endolysosome I_{TRPML1} that persisted upon washout of the drugs in EGFP-TRPML1-transfected COS1 cells.

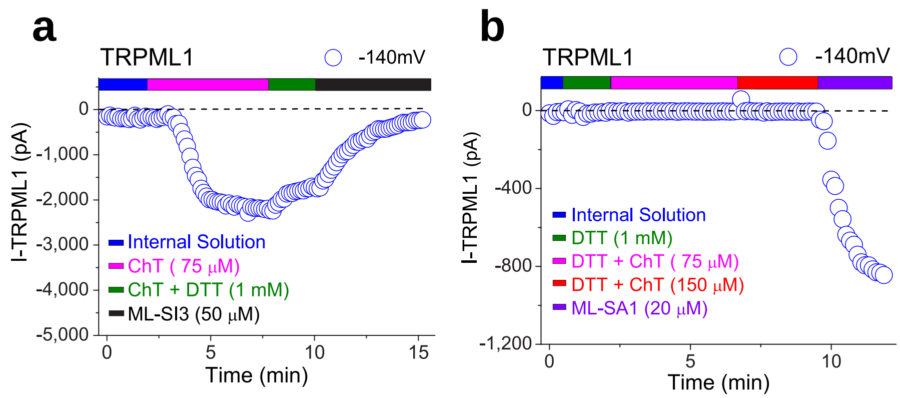

Supplementary Figure 2. Redox dependence of ChT-induced activation of TRPML1.
(a) Acute application of DTT (1 mM) only slightly reduced ChT-activated whole-endolysosome I_{TRPML1} in EGFP-TRPML1-transfected COS1 cells. **(b)** DTT pretreatment for 2 min prevented I_{TRPML1} activation by ChT, but not ML-SA1.

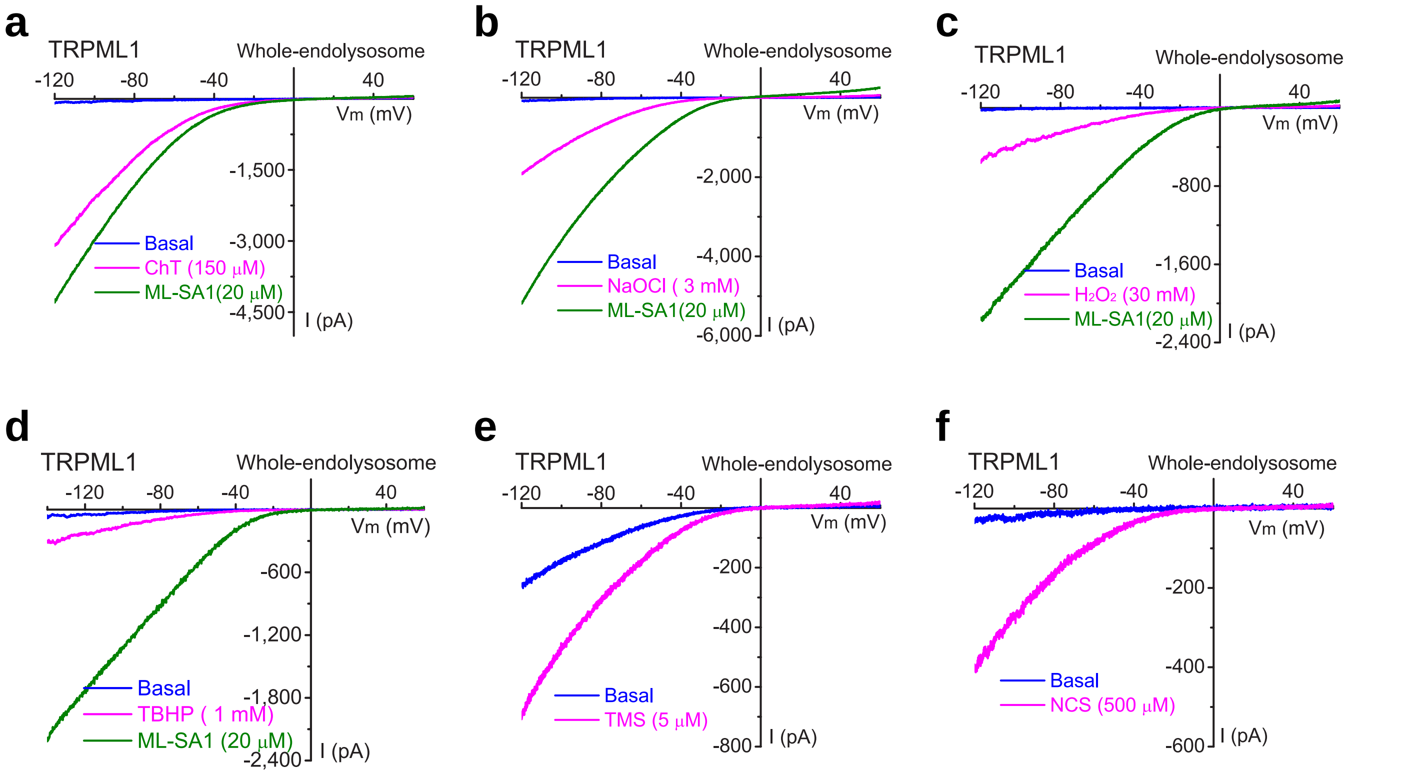

Supplementary Figure 3. Activation of I_{TRPML1} by oxidants.
(a-f) Whole-endolysosome I_{TRPML1} was activated by ChT (150 μM; **a**), NaOCl (3 mM; **b**), H_2O_2 (30 mM; **c**), tert-butyl hydroperoxide (TBHP, 1 mM; **d**), thimerosal (TMS, 50 μM; **e**), and N-chlorosuccinimide (NCS, 500 μM; **f**).

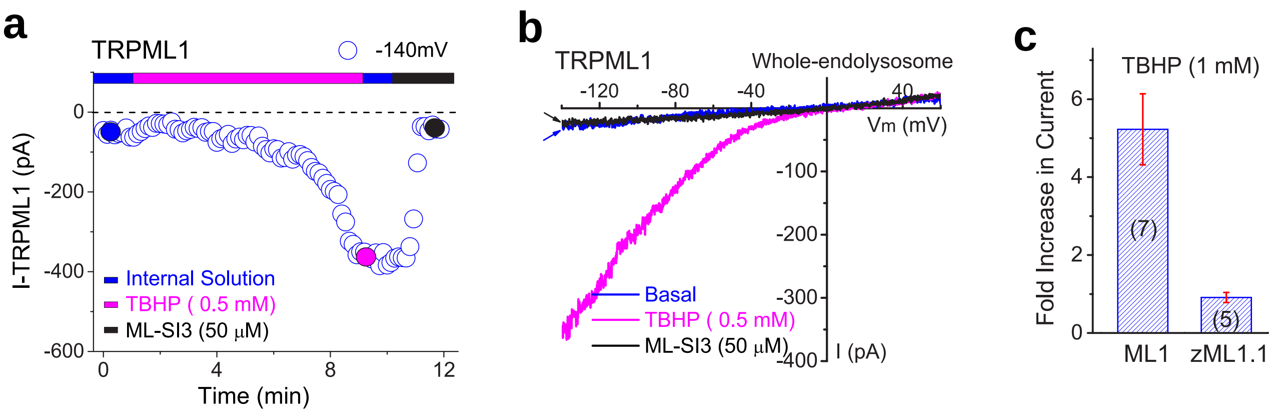

Supplementary Figure 4. Activation of mouse TRPML1 by low dose TBHP.
(a) Whole-endolysosomal mTRPML1 currents were activated by 0.5 mM TBHP, and the activation was inhibited by ML-SI3. **(b)**The representative traces of basal, TBHP-activated, and ML-SI3-inhibited mTRPML1 currents at time points indicated in (**a**). **(c)** Mouse TRPML1, but not Zebrafish TRPML1.1 was activated by TBHP (1 mM). Means are shown with SEM; n= 7 and 5 for I_{TRPML1} and $I_{zTRPML1.1}$, respectively.

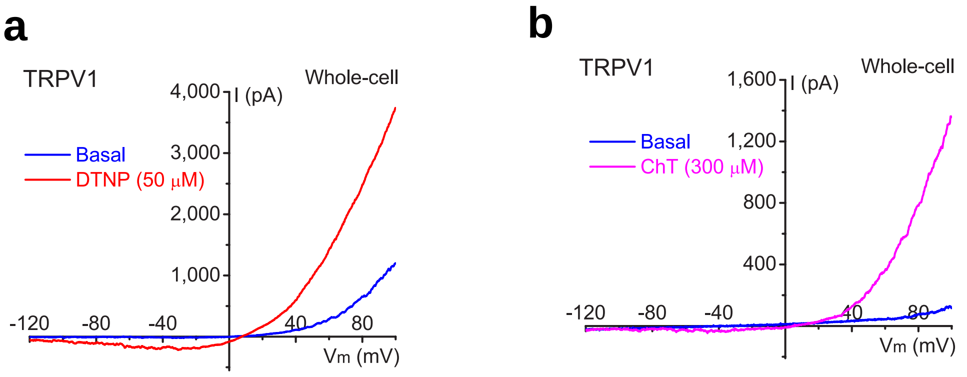

Supplementary Figure 5. Activation of TRPV1 by DTNP and ChT.
Whole-cell TRPV1 currents were activated by DTNP (**a**) and ChT (**b**) in TRPV1-expressing HEK293 cells.

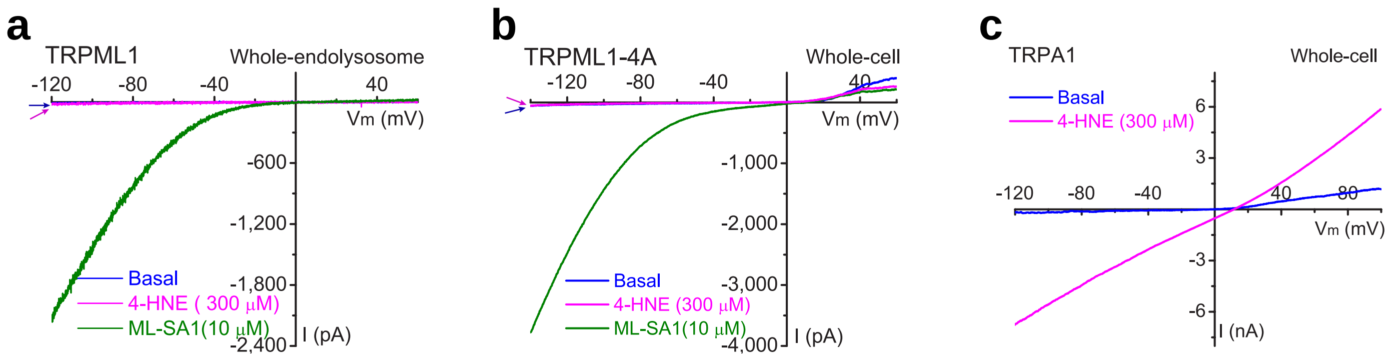

Supplementary Figure 6. TRPML1 is not activated by 4-HNE, a reactive intermediate of lipid peroxidation.
(a, b) 4-HNE failed to activate I_{TRPML1} in both whole-endolysosome and whole-cell patches. **(c)** TRPA1 was readily activated by 4-HNE.

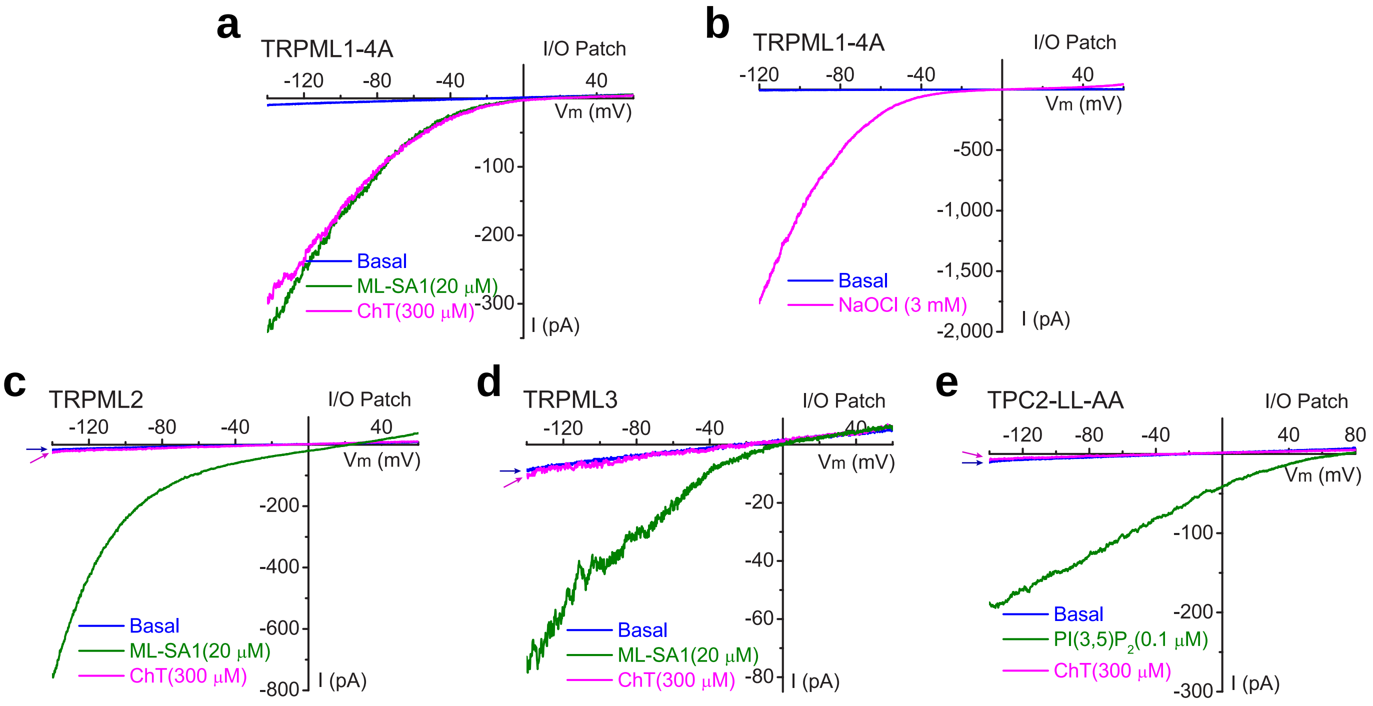

Supplementary Figure 7. Oxidants specifically activate TRPML1, but not other related channels in inside-out patches.
(a, b) Activation of $I_{TRPML-4A}$ by ChT (**a**) and NaOCl (**b**) in inside-out patches from TRPML1-4A-expressing HEK293 cells. **(c-e)** A high dose (300 μM) of ChT failed to activate I_{TRPML2} (**c**), I_{TRPML3} (**d**), or $I_{TPC2-LL-AA}$ (a surface-expressing mutant TPC2) (**e**) in inside-out patches.

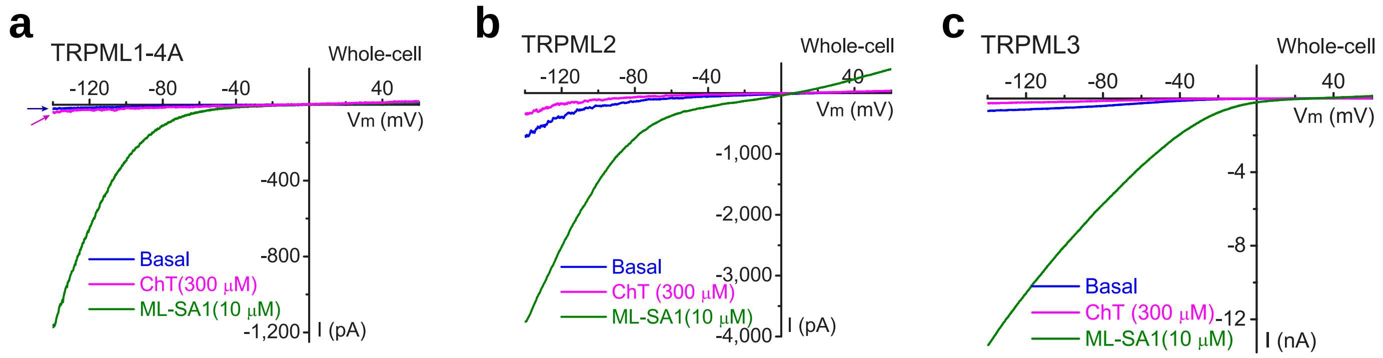

Supplementary Figure 8. Oxidants do not activate $I_{TRPML1-4A}$ in the whole-cell configuration.
(a) ChT failed to activate $I_{TRPML1-4A}$ in TRPML1-4A-expressing HEK293 cells. ML-SA1 activated whole-cell $I_{TRPML1-4A}$ readily. **(b)** ML-SA1, but not ChT, activated whole-cell I_{TRPML2} in TRPML2 -expressing HEK293 cells. **(c)** ML-SA1, but not ChT, activated whole-cell I_{TRPML3} in TRPML3-expressing HEK293 cells.

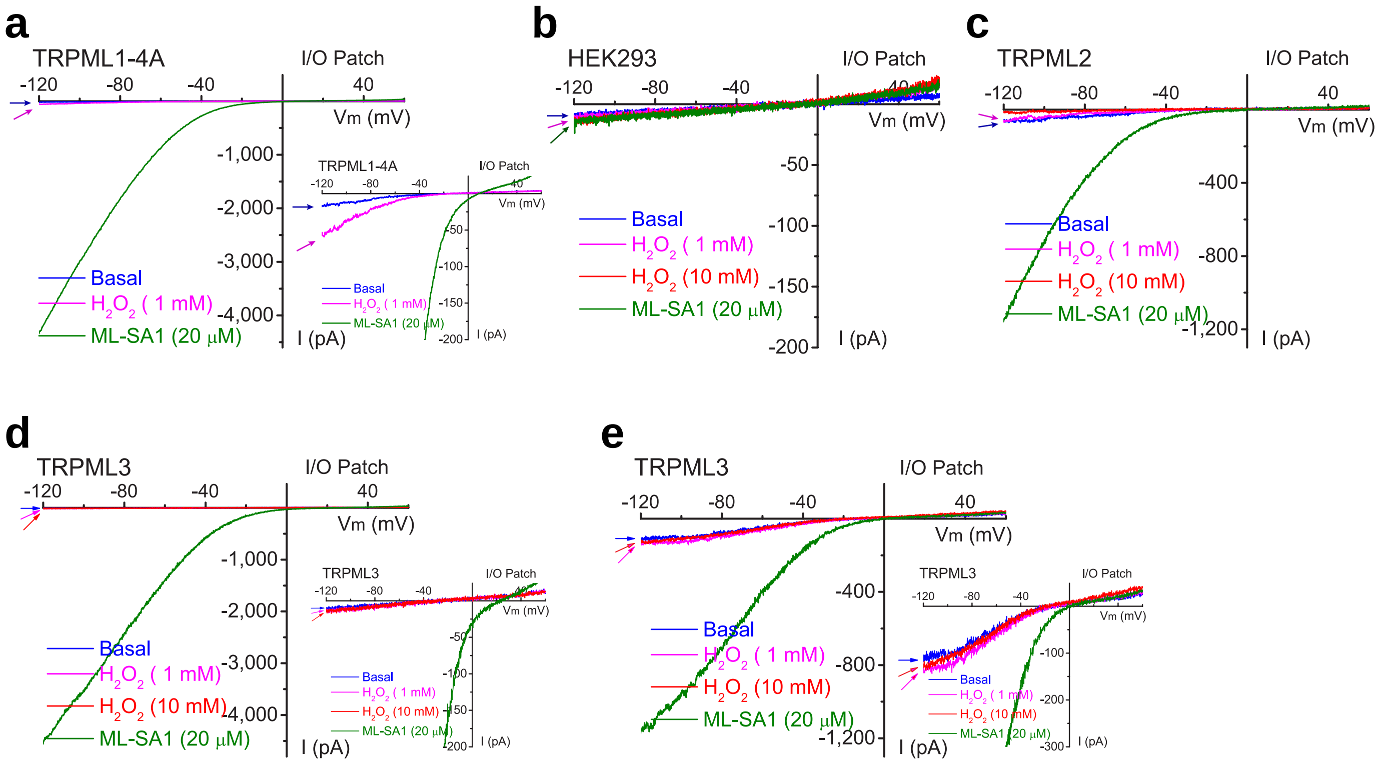

Supplementary Figure 9. H_2O_2 specifically activates $I_{TRPML1\text{-}4A}$ in inside-out patches.
(a) Activation of $I_{TRPML1\text{-}4A}$ by H_2O_2 (1 mM) and ML-SA1 in inside-out patches from EGFP-TRPML1-4A -expressing HEK293 cells. In comparison with ML-SA1, low dose of H_2O_2 only had subtle activation effects on $I_{TRPML1\text{-}4A}$. The inset showed the expanded view of H_2O_2-activation of $I_{TRPML1\text{-}4A}$. **(b-e)** A high dose (10 mM) of H_2O_2 failed to activate endogenous currents (**b**), I_{TRPML2} (**c**), I_{TRPML3} (**d**), or I_{TRPML3} with basal activity (**e**) in inside-out patches from HEK293 cells that were transfected with EGFP-C1 vector, mCherry-TRPML2, and EGFP-TRPML3, respectively.

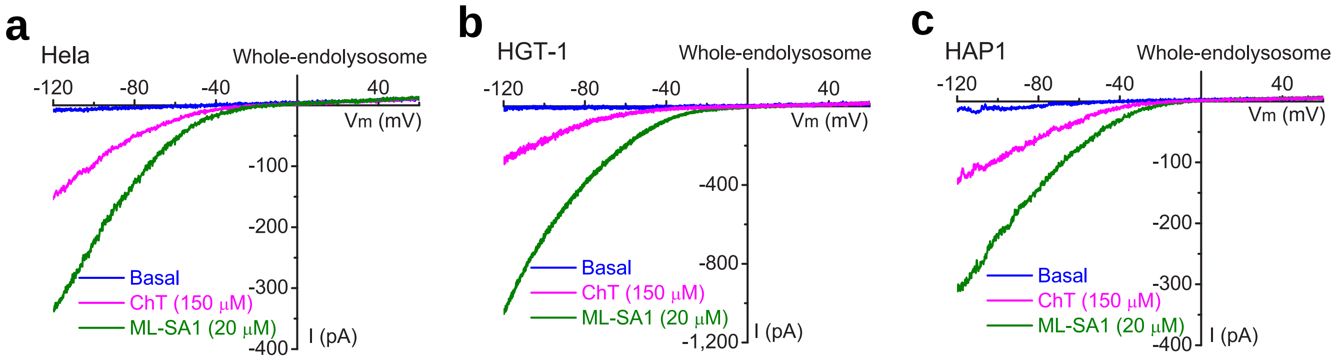

Supplementary Figure 10. Oxidants activate endogenous lysosomal TRPML1.
ChT activated whole-endolysosome I_{TRPML1} in non-transfected HeLa (**a**), HGT-1 (**b**, a parietal cell line), and HAP1(**c**, a haploid human cell line) cells.

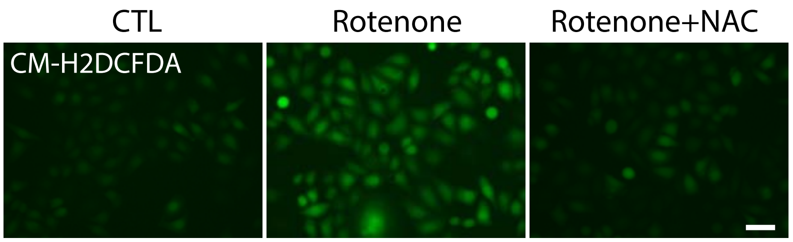

Supplementary Figure 11. Rotenone treatment increases ROS levels in HeLa cells.
Rotenone (10 μM) treatment for 1 h increased the fluorescence intensity of CM-H2DCFDA compared with the DMSO-treated control group (CTL). The increase was inhibited by co-application of NAC. Scale bar = 50 μm.

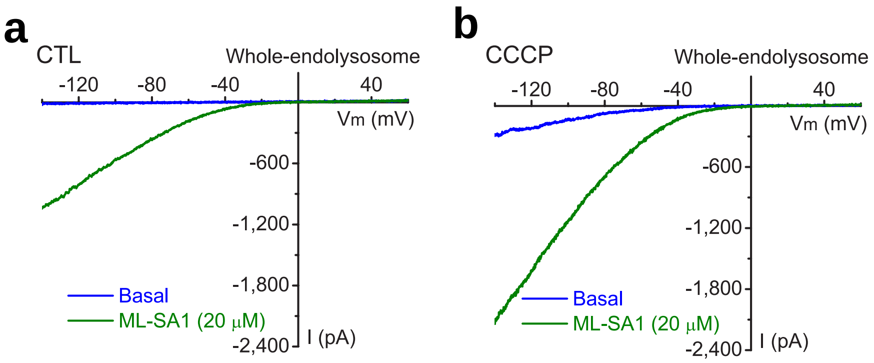

Supplementary Figure 12. Mitochondrial ROS increase endogenous TRPML1 currents.
CCCP pretreatment (10 μM; **b**) for 1 h increased endogenous whole-endolysosome I_{TRPML1} in HEK293 cells compared with DMSO controls (**a**).

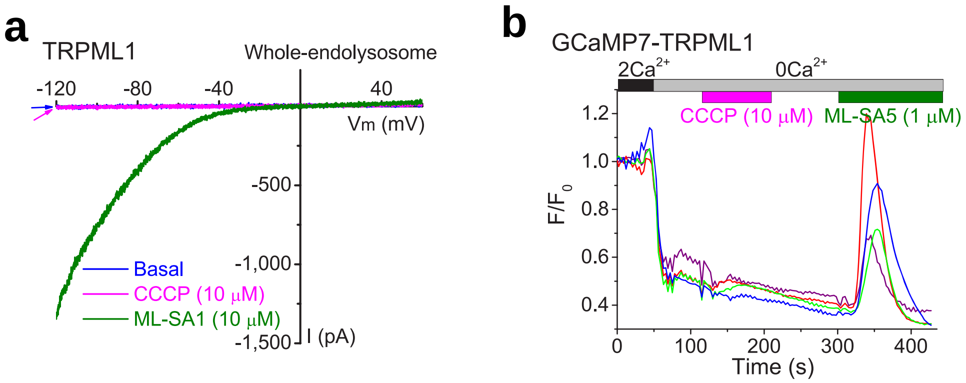

Supplementary Figure 13. CCCP did not activate TRPML1 directly.
(a) Whole-endolysosome I_{TRPML1} was not sensitive to acute, bath application of CCCP (10 μM). **(b)** In GCaMP7-TRPML1-expressing HeLa cells, CCCP did not evoke Ca^{2+} release from intracellular Ca^{2+} stores. ML-SA5 readily elicited rapid and robust Ca^{2+} responses in the same cells.

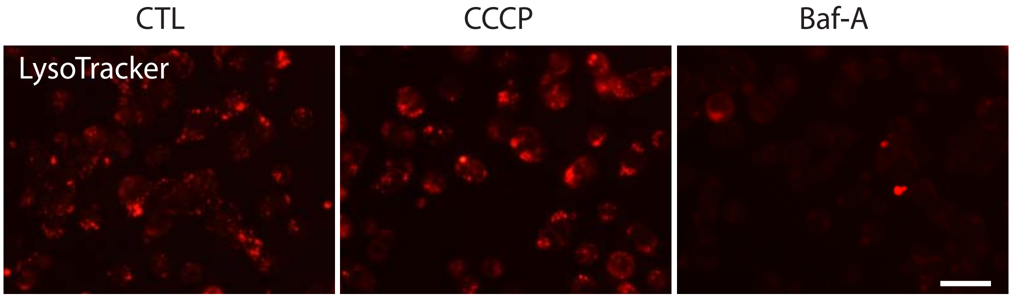

Supplementary Figure 14. CCCP did not change lysosomal pH.
CCCP (20 μM) treatment for 1 h did not reduce LysoTracker staining. In contrast, Bafilomycin A1 (Baf-A, 1 μM) treatment for 1 h abolished LysoTracker staining. Scale bar = 50 μm.

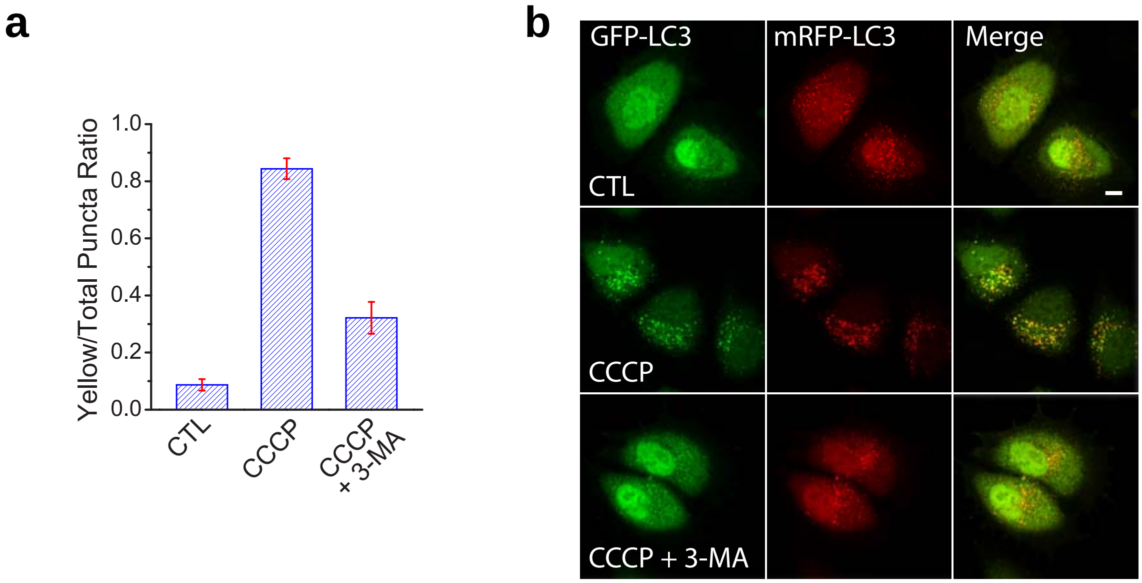

Supplementary Figure 15. CCCP treatment promotes autophagosome formation.
(a-b) CCCP pretreatment (5 μM) for 3 h increased the number of $GFP^{+}mRFP^{+}$ LC3 puncta in GFP-mRFP-LC3 stable cells, and the increase was blocked by co-application of 3-MA (5 mM). Data are presented as mean ± SEM (from multiple randomly-selected images). Scale bar = 10 μm.

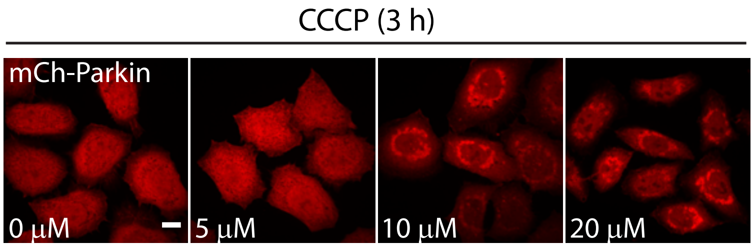

Supplementary Figure 16. Dose dependence of CCCP-induced accumulation of PARKIN puncta. High concentrations (10–20 μM), but not a low concentration (5 μM) of CCCP induced PARKIN puncta formation in HeLa cells stably expressing mCherry-Parkin. Scale bar = 10 μm.

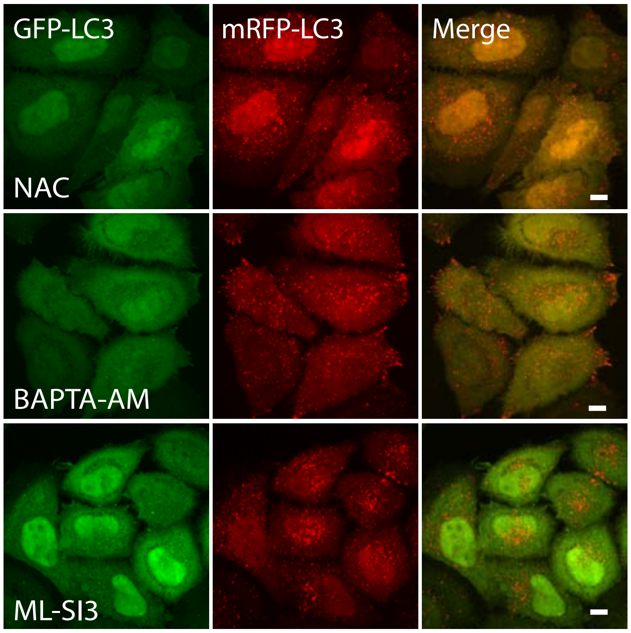

Supplementary Figure 17. NAC, BAPTA-AM, or ML-SI3 alone does not induce autophagy. NAC (5 mM), BAPTA-AM (10 μM), or ML-SI3 (10 μM) alone did not increase the number of GFP^+ $mRFP^+$ LC3 puncta in GFP-mRFP-LC3 stable cells. Scale bar = 10 μm.

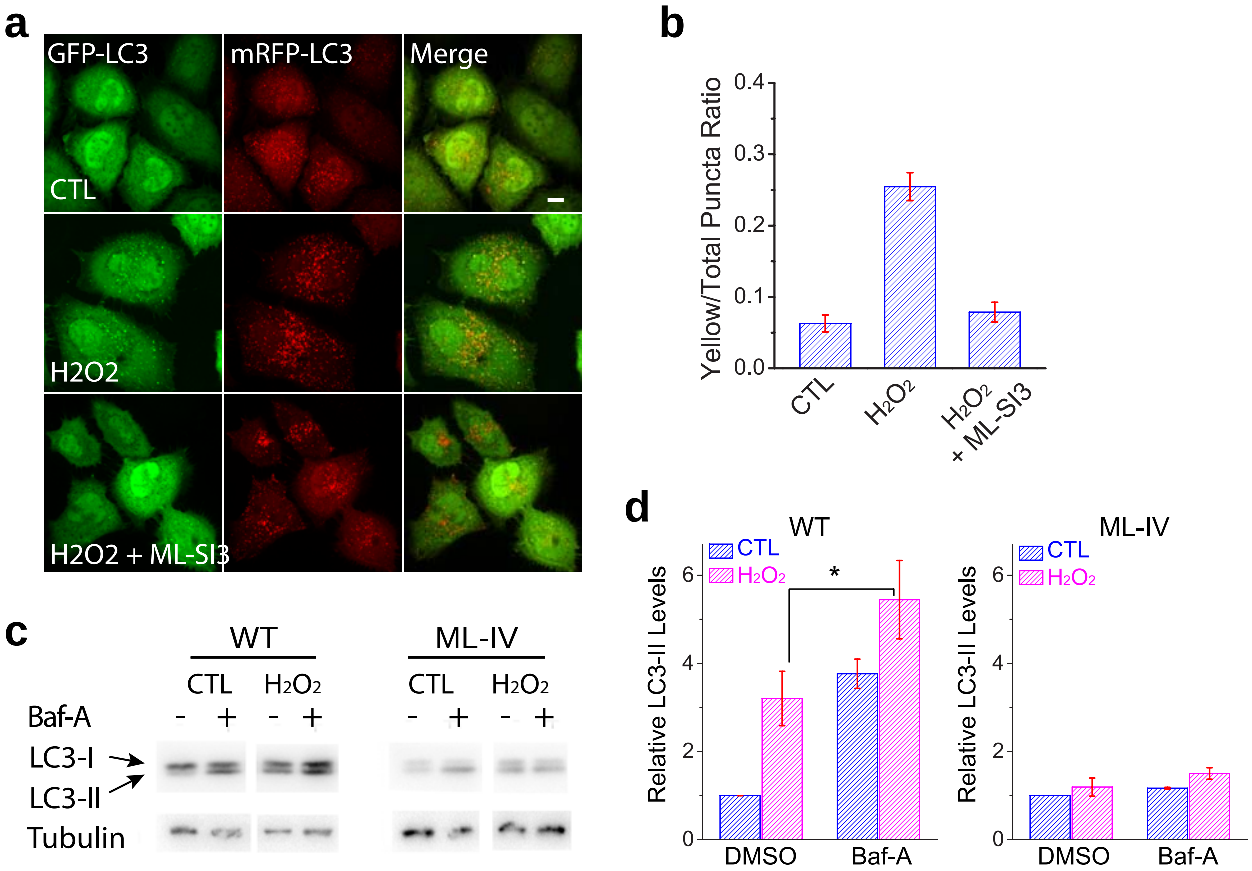

Supplementary Figure 18. H_2O_2 promotes autophagosome formation through TRPML1 activation.

(a) H_2O_2 treatment (100 µM for 3 h) increased the number of GFP mRFP LC3 puncta in HeLa cells, and the increase was inhibited by co-application of ML-SI3.Scale bar = 10 µm. **(b)** Quantification of **(a)** from more than 50 cells. Means are shown with SEM. **(c)** LC3-II levels were increased by H_2O_2 treatment in WT, but not ML-IV fibroblasts. **(d)** Quantification of **(c)** from three independent experiments. Data are presented as mean ± SEM. *$P < 0.05$, paired *t*-test.

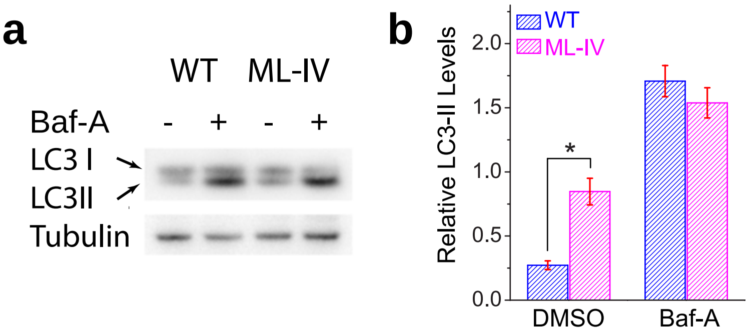

Supplementary Figure 19. Elevated LC3-II levels in ML-IV fibroblasts.
(a) Basal levels of LC3-II were higher in ML-IV fibroblasts compared with WT fibroblasts. Blockage of lysosome function using Bafilomycin A1 increased LC3-II levels in both WT and ML-IV cells.
(b) Quantification of **(a)** from three independent experiments (mean ± SEM); $*P < 0.05$, paired *t*-test.

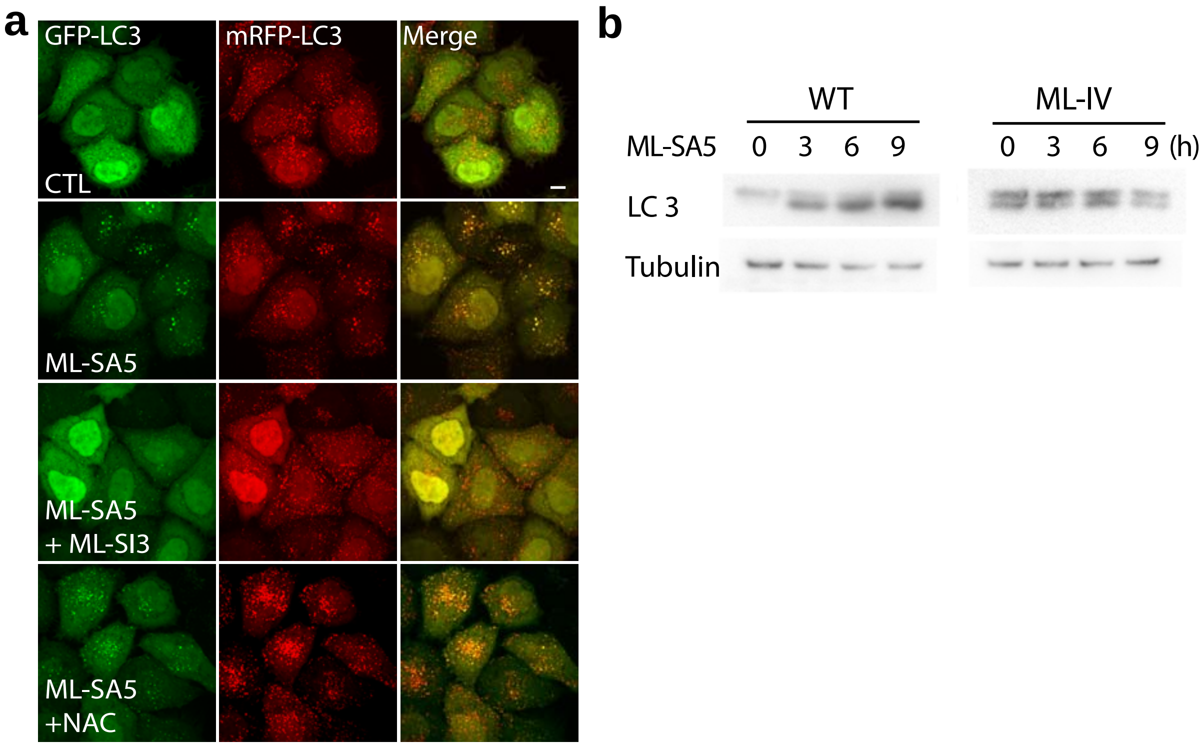

Supplementary Figure 20. Activation of TRPML1 by ML-SA5 is sufficient to induce autophagy.
(a) Effects of ML-SA5 on GFP^+ LC3 puncta formation in the presence and absence of ML-SI3 or NAC. Scale bar = 10 µm. **(b)** Time dependence (0 h, 3 h, 6 h, and 9 h) of ML-SA5 (1 µM) treatment on LC3-II accumulation in human WT and ML-IV fibroblasts.

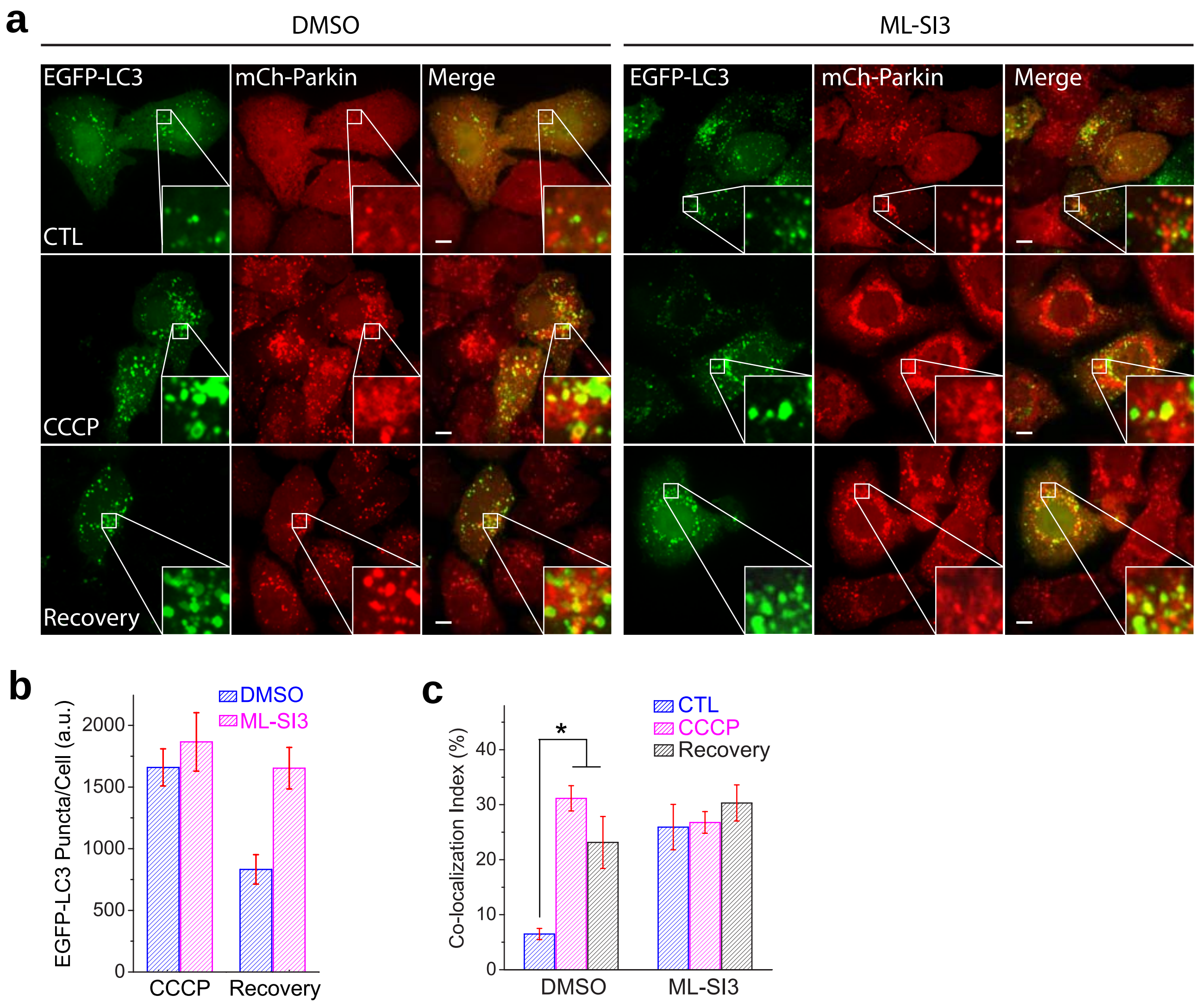

Supplementary Figure 21. CCCP-induced mitophagy is inhibited by ML-SI3.
(a) In GFP-LC3-expressing PARKIN stable cells, the co-localization (yellow) of LC3 (green) with PARKIN (red) puncta was enhanced by CCCP (10 μM, 3h). The high level of co-localization persisted even after washout of CCCP (recovery phase). Co-application of ML-SI3 abolished the co-localization. Scale bar = 10 μm. **(b, c)** LC3 puncta (**b**) and LC3-PARKIN co-localization (**c**) were quantified from multiple randomly-selected representative images. Co-localization index was defined as the ratio of LC3-PARKIN co-aggregates (yellow puncta) to PARKIN aggregates (red puncta). Data are presented as mean ± SEM. *$P < 0.05$, ANOVA.

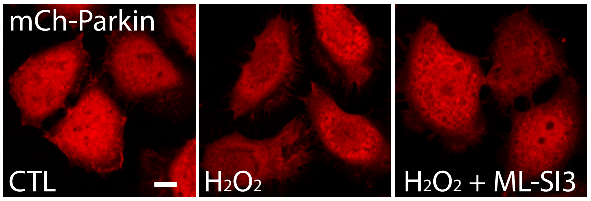

Supplementary Figure 22. H_2O_2 does not induce PARKIN aggregation.
Treatment of HeLa cells stably expressing mCherry-Parkin with 100 μM H_2O_2 for 3 h did not lead to detectable accumulation of PARKIN puncta. Scale bar = 10 μm.

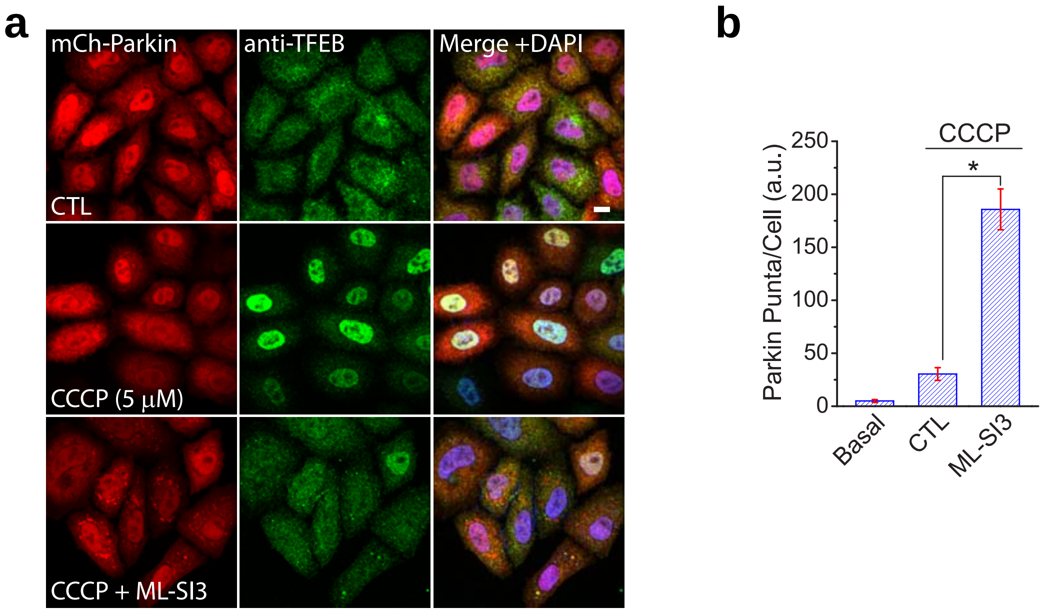

Supplementary Figure 23. Inhibition of TRPML1 blocks TFEB nuclear translocation and promotes CCCP-induced mitochondrial damage.
(a) In PARKIN stable cells, CCCP treatment (5 μM for 3 h) led to minimal PARKIN aggregation, but strong TFEB nuclear translocation. Co-application of ML-SI3 enhanced PARKIN accumulation but inhibited TFEB activation. Scale bar = 10 μm. **(b)** Quantification of PARKIN puncta shown in **(a)** (>50 cells per experimental condition; mean ± SEM). $^*P < 0.05$, ANOVA.

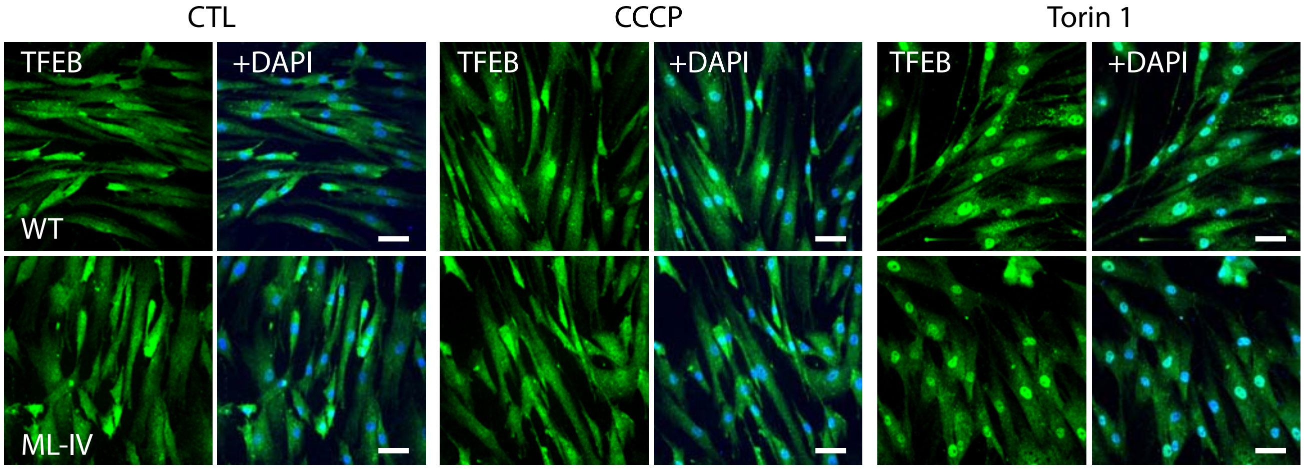

Supplementary Figure 24. CCCP induced TFEB nuclear translocation in WT but not ML-IV human fibroblasts.
Nuclei were counterstained with DAPI. Scale bar = 50 µm.

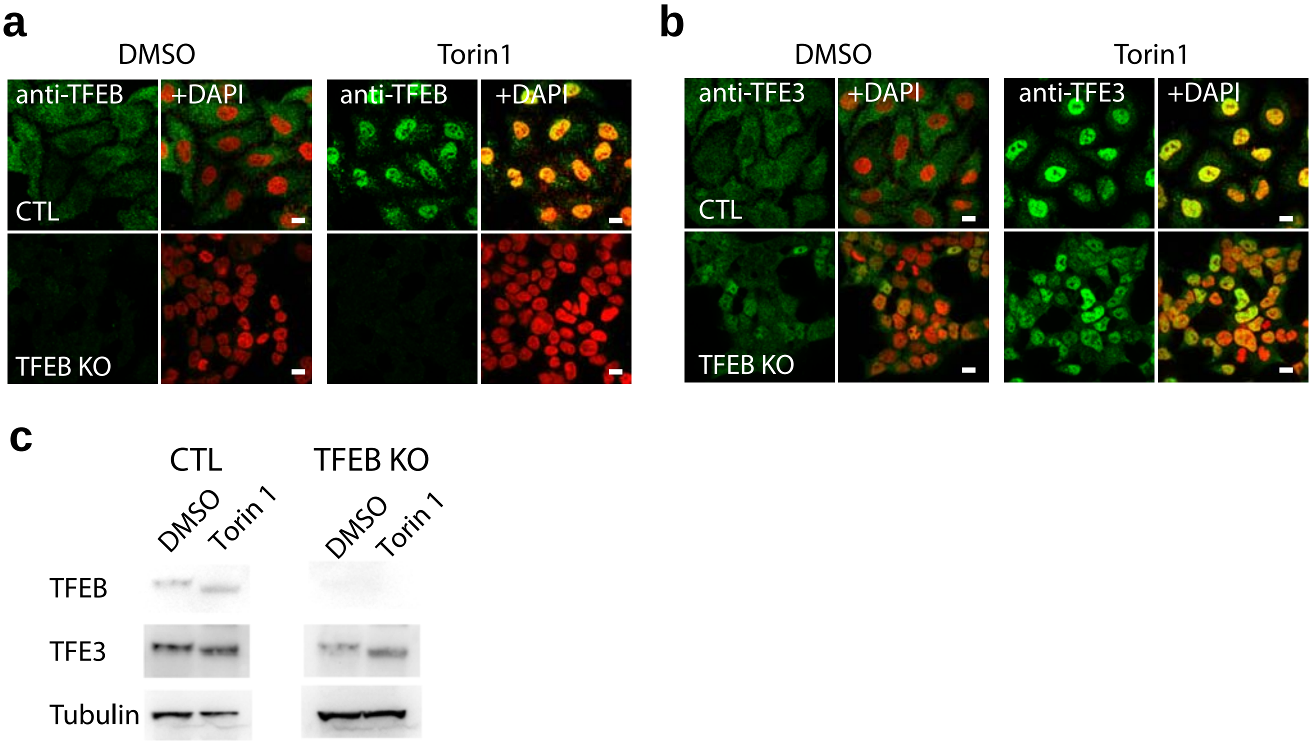

Supplementary Figure 25. Specificity of TFEB antibodies.
(a) The immunoreactivity of TFEB, but not related TFE3 (**b**), was abolished in TFEB KO HeLa cells. Torin1 treatment induced nuclear translocation of TFE3. Nuclei were counterstained with DAPI (pseudo-colored in green). Scale bar = 10 µm. **(c)** TFEB proteins were not detectable in TFEB KO cells. Note that inhibition of mTOR by Torin1 induced a size shift of TFE3 proteins in both WT and TFEB KO cells.

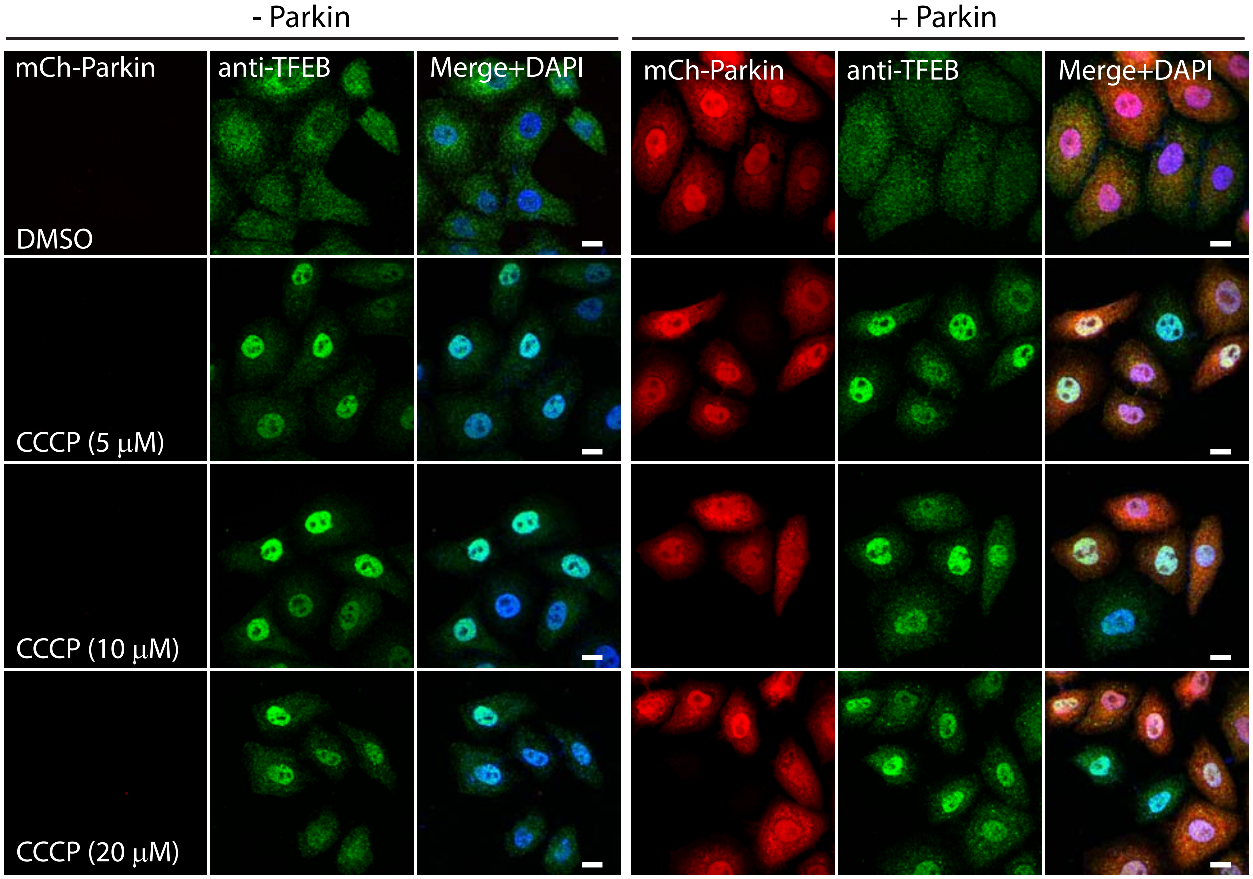

Supplementary Figure 26. CCCP-induced TFEB translocation is independent of PARKIN expression.

CCCP (5 μM, 10 μM, or 20 μM) treatment for 1 h induced TFEB translocation in HeLa cells with or without stable expression of mCherry-Parkin. Nuclei were counterstained with DAPI. Scale bar = 10 μm.

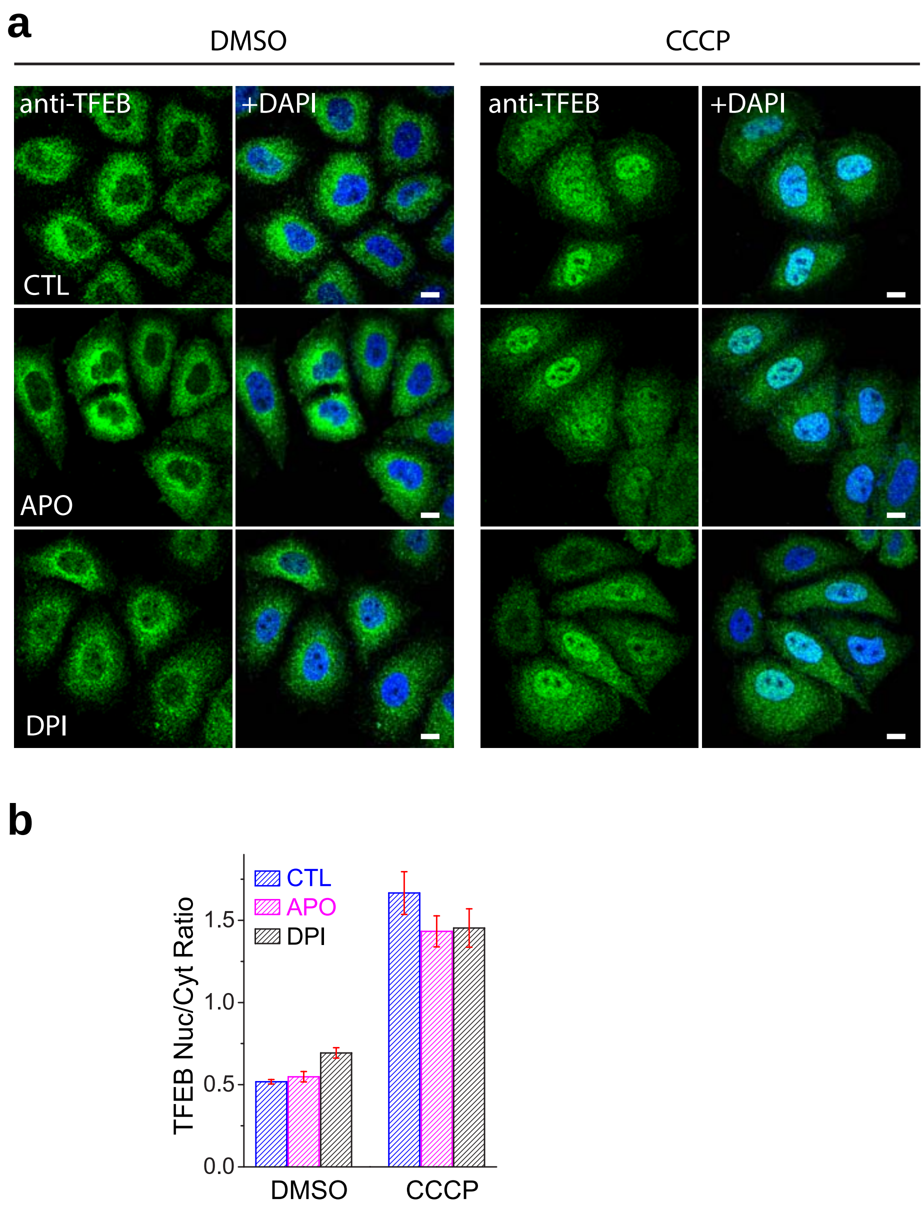

Supplementary Figure 27. NOX inhibitors did not block CCCP-induced TFEB nuclear translocation. (a) Application of apocynin (APO, 100 µM) or diphenyleneiodonium (DPI, 2.5 µM) did not significantly affect TFEB nuclear translocation induced by CCCP. Nuclei were counterstained with DAPI. Scale bar = 10 µm. **(b)** Quantification of **(a)** from at least 30 cells for each experimental condition. Means are shown with SEM; *$P < 0.05$, ANOVA.

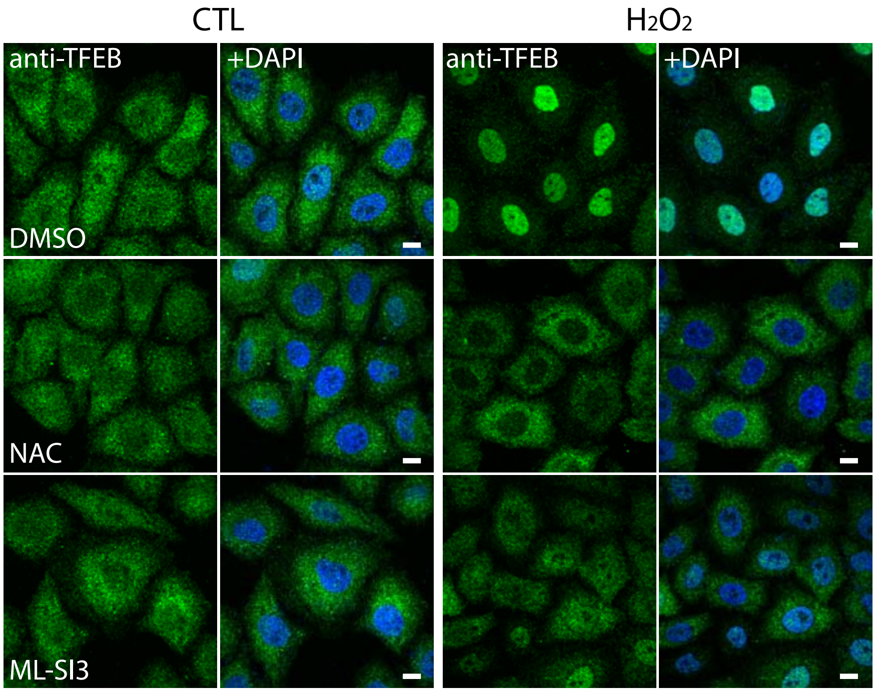

Supplementary Figure 28. H_2O_2 induced TFEB nuclear translocation.
TFEB nuclear translocation induced by H_2O_2 (50 µM) was effectively blocked by NAC or ML-SI3. Nuclei were counterstained with DAPI. Scale bar = 10 µm.

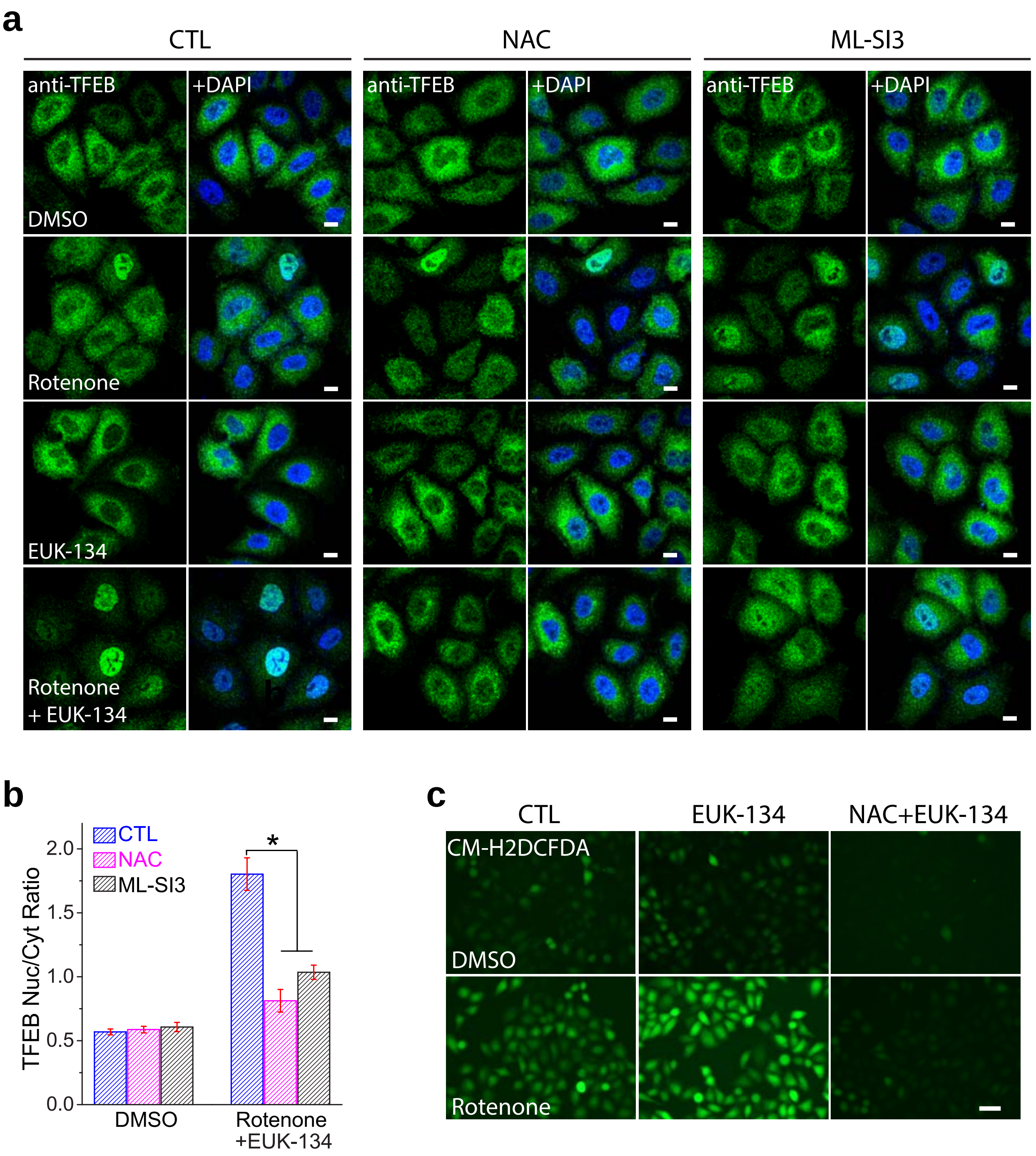

Supplementary Figure 29. Rotenone-induced ROS generation causes TFEB nuclear translocation. **(a)** Although rotenone alone only induced mild TFEB nuclear translocation, co-application of EUK-134 (a synthetic dismutase/catalase mimetic) markedly enhanced TFEB activation. Both NAC and ML-SI3 blocked rotenone-induced TFEB activation. Nuclei were counterstained with DAPI. Scale bar = 10 µm. **(b)** Quantification of **(a)** from at least 50 cells for each experimental condition. Mean ± SEM are shown; **P* < 0.05, ANOVA. **(c)** ROS levels (detected with CM-H2DCFDA) were augmented by rotenone (10 µM, 2h), which was further increased by co-application of EUK-134 (50 µM). NAC abolished CM-H2DCFDA fluorescence in cells treated with rotenone alone, or together with EUK-134. Scale bar = 50 µm.

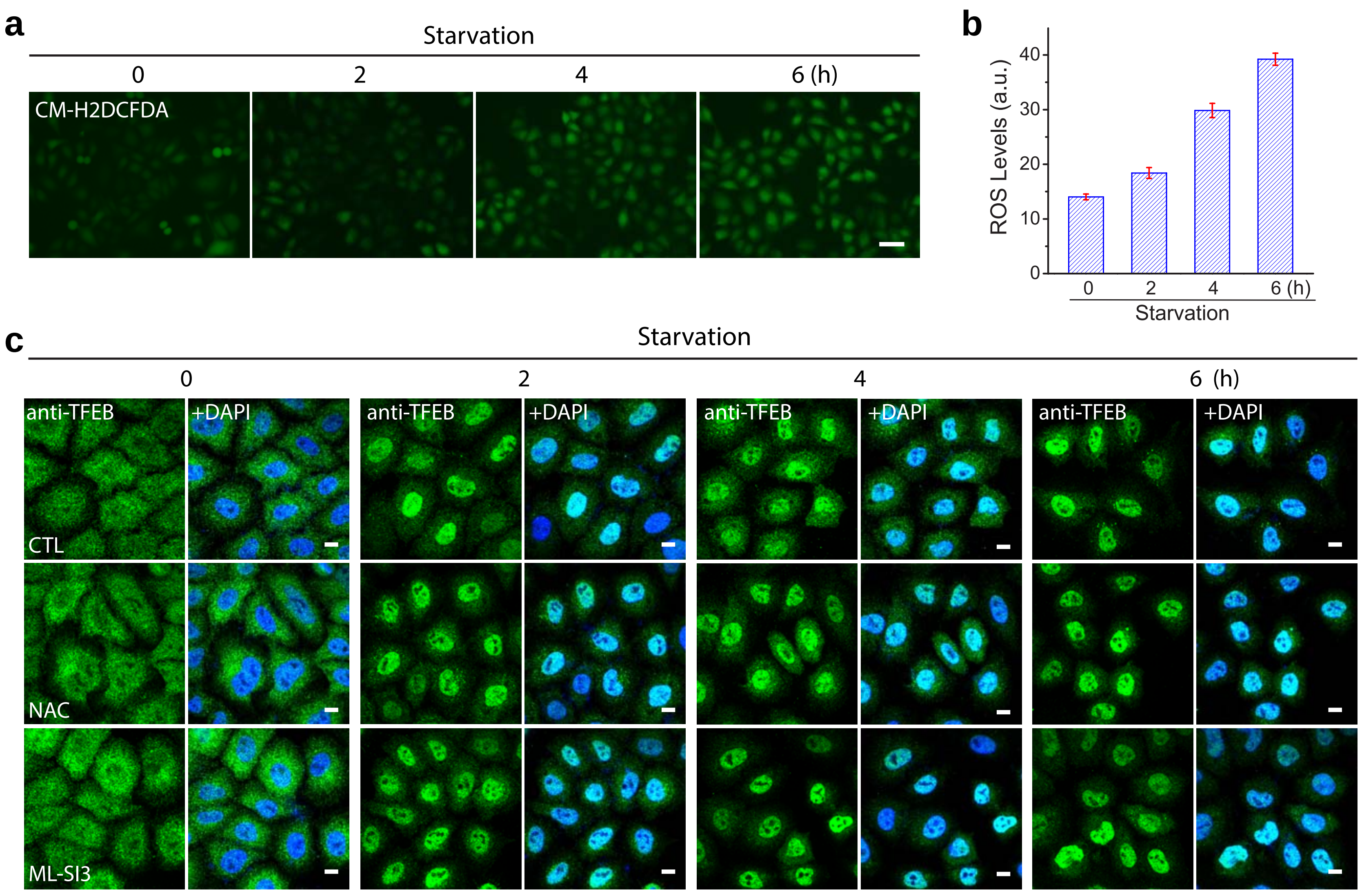

Supplementary Figure 30. Starvation-induced TFEB nuclear translocation does not require ROS or TRPML1.
(a) Starvation-induced time-dependent increment in ROS levels as quantified in **(b)**(shown as mean ± SEM from at least 100 cells for each condition). Starvation is induced by deprivation of both FBS and amino acids in the culture media. Scale bar = 50 μm.
(c) Neither NAC not ML-SI3 diminished TFEB activation induced by short term (2, 4, 6 h) starvation. Nuclei were counterstained with DAPI. Scale bar = 10 μm.

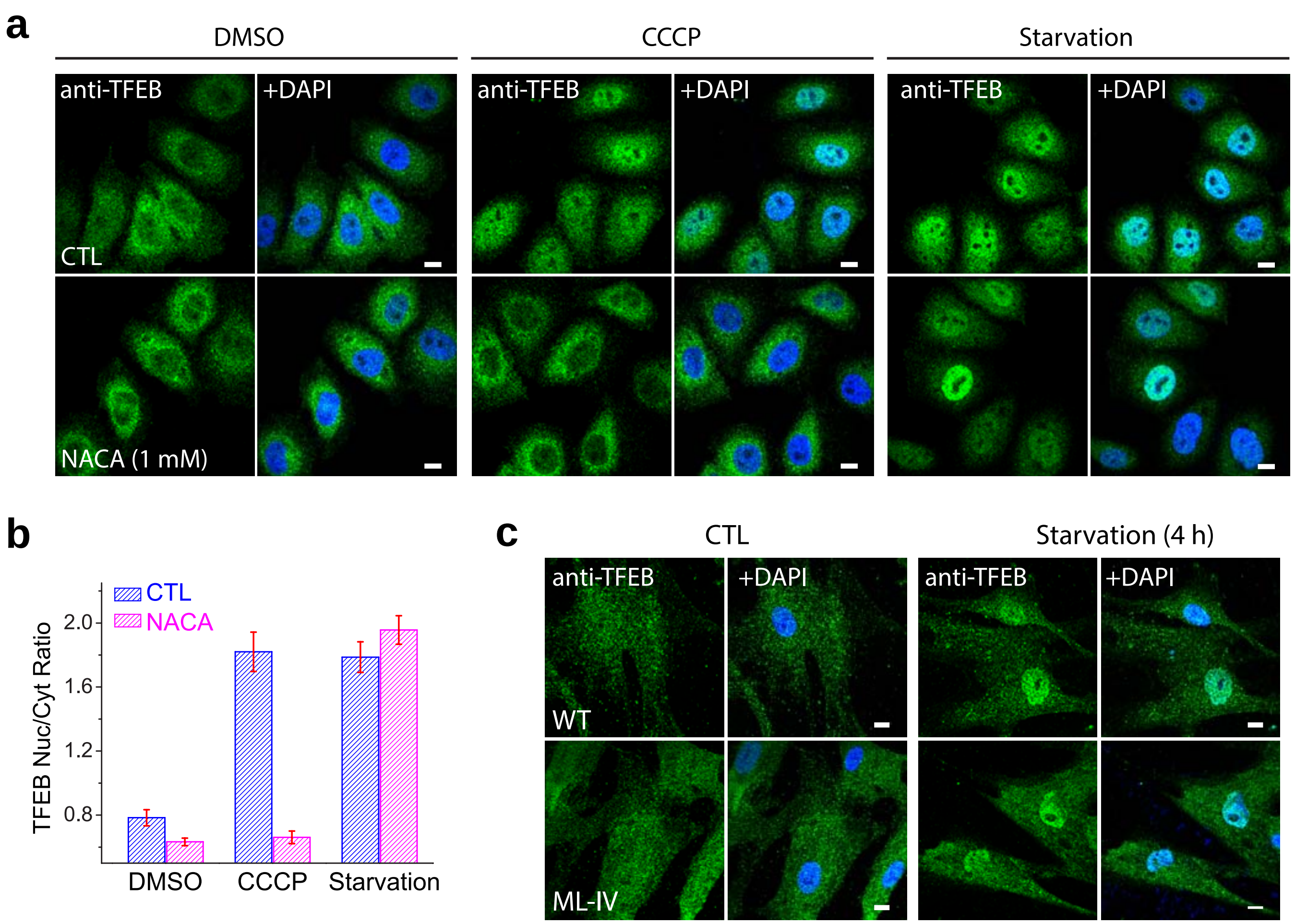

Supplementary Figure 31. Starvation induced TFEB nuclear translocation in the presence of NACA and in ML-IV fibroblasts.

(a) NACA, a potent NAC derivative, inhibited CCCP- but not starvation (1h)-induced TFEB activation. Scale bar = 10 μm. **(b)** Average effects of NACA. Mean are shown with SEM from ~40 randomly selected cells per experimental condition. **(c)** Starvation (4h) activated TFEB in both WT and ML-IV human fibroblasts. Nuclei were counterstained with DAPI. Scale bar = 10 μm.

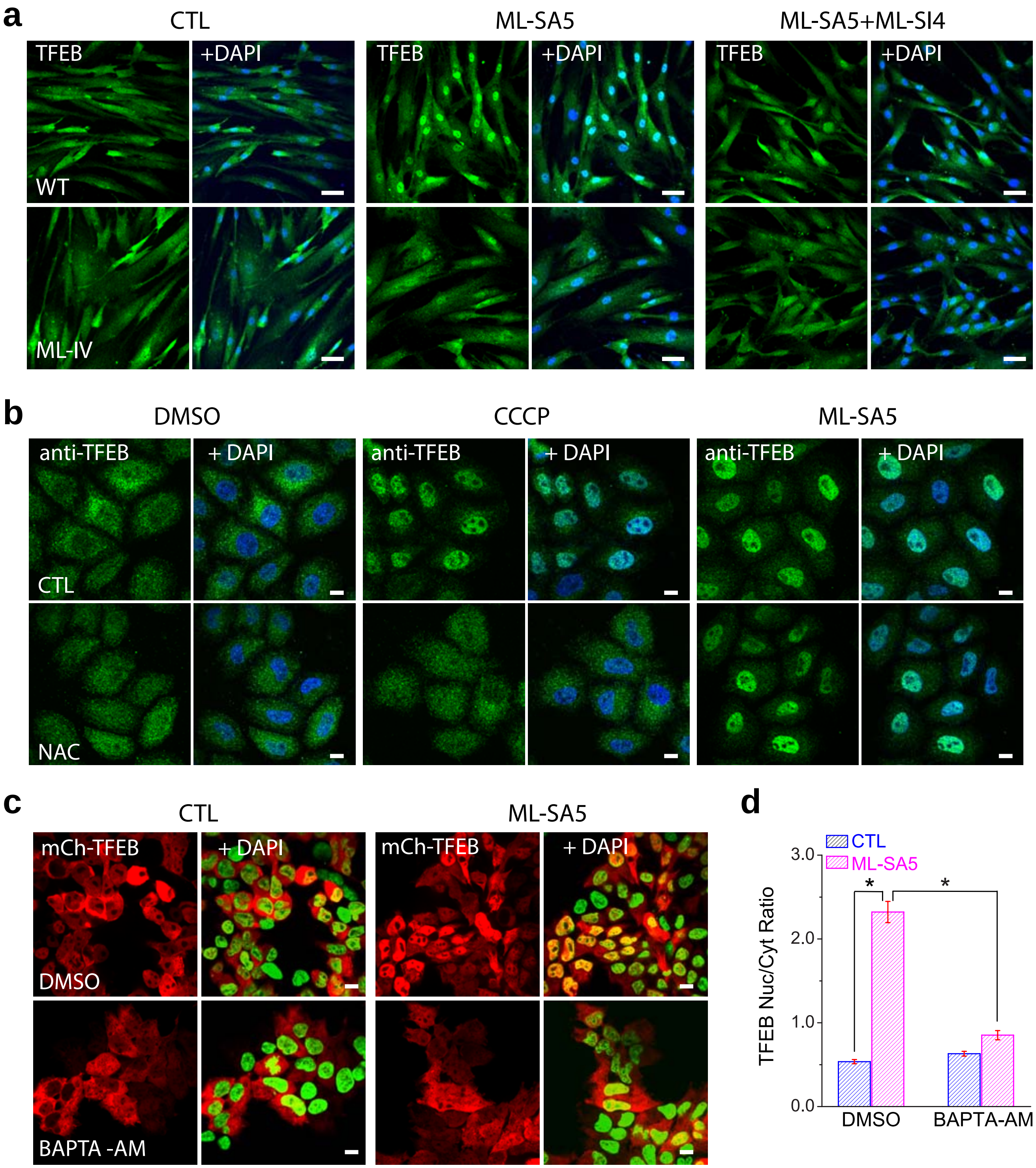

Supplementary Figure 32. ML-SA5-induced TFEB nuclear translocation in WT cells is blocked by ML-SI4 or BAPTA-AM but not by NAC.

(a) ML-SA5 induced TFEB nuclear translocation in WT but not ML-IV human fibroblasts, which was effectively blocked by ML-SI4. Scale bar = 50 μm. **(b)** CCCP-induced, but not ML-SA5–induced, TFEB nuclear translocation was blocked by NAC in HeLa cells. Scale bar = 10 μm. **(c)** ML-SA5 (1 μM for 1 h) -induced TFEB nuclear translocation was blocked by BAPTA-AM in TFEB stable cells. Nuclei were counterstained with DAPI (pseudo-colored with green). Scale bar = 10 μm. **(d)** Quantification of TFEB activation by ML-SA5, shown as mean ± SEM (>100 randomly-selected cells per experimental condition); *$P < 0.05$, ANOVA.

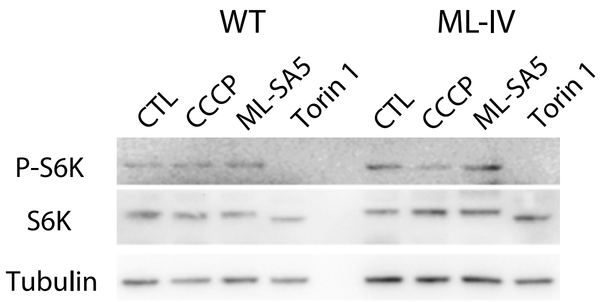

Supplementary Figure 33. mTOR activity was not reduced by CCCP or ML-SA5 treatment.
The activity of mTOR was assessed by phosphorylated S6K level assays in WT and ML-IV fibroblasts. Torin1 was used as a positive control.

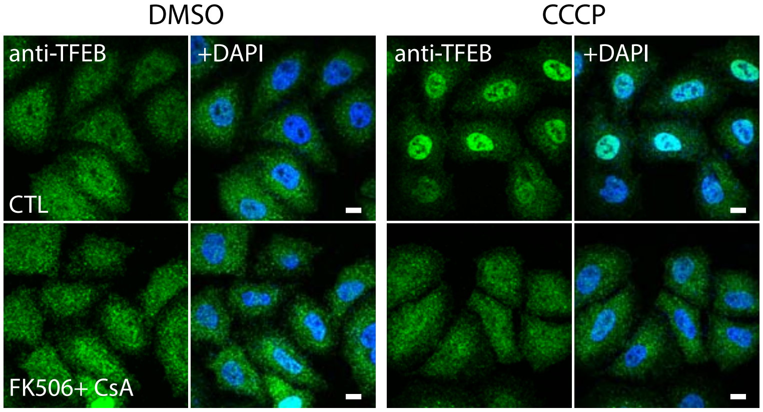

Supplementary Figure 34. Calcineurin inhibitors block CCCP-induced TFEB translocation.
CCCP (5 μM for 1 h)-induced TFEB nuclear translocation was blocked by co-application of FK506 (5 μM) and CsA (10 μM) in HeLa cells. Nuclei were counterstained with DAPI. Scale bar = 10 μm.

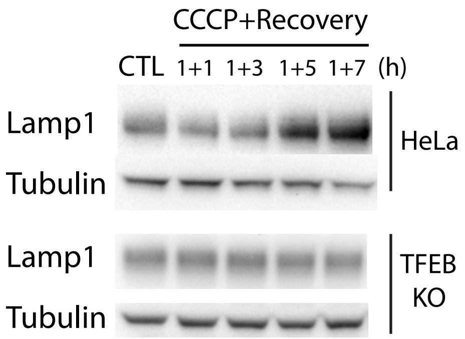

Supplementary Figure 35. CCCP treatment increases Lamp1 expression in WT, but not TFEB KO HeLa cells.
Time course of Lamp1 expression levels revealed by western blot analysis after brief (5 μM, 1 h) CCCP pre-treatment in WT (upper panel) and TFEB KO (lower panel) HeLa cells.

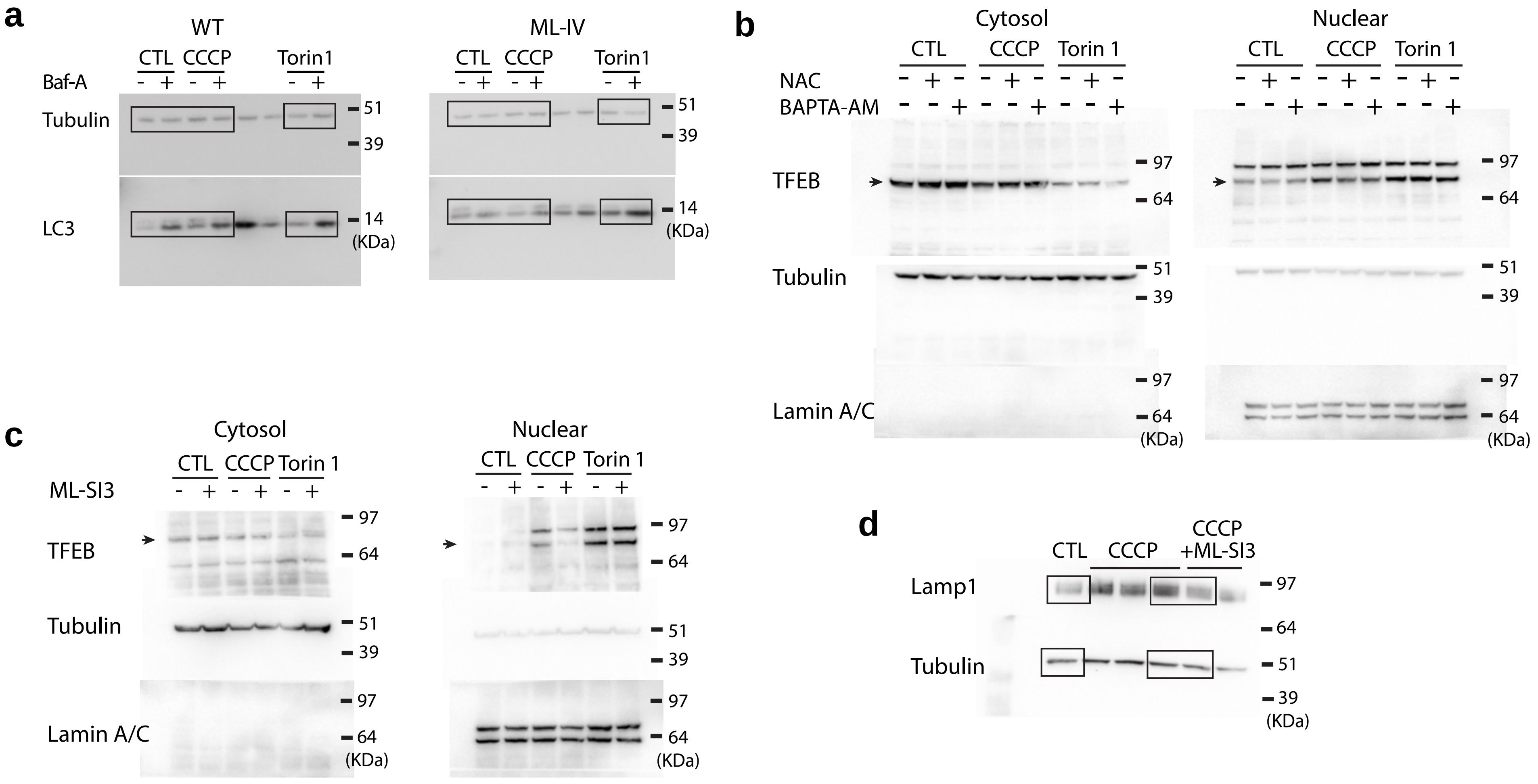

Supplementary Figure 36. Full blot images related to main fiures.
(a) Full blots related to Figure 3d. Protein bands shown in Fig. 3d as indicated by boxes. **(b)** Full blots related to Figure 5c. TFEB is indicated by an arrow. **(c)** Full blots related to Figure 5e. TFEB is indicated by an arrow. **(d)** Full blots related to Figure 6a. Protein bands shown in Fig. 6a as indicated by boxes.
